# Nature redux: interrogating biomorphism and soft robot aesthetics through generative AI

**DOI:** 10.3389/frobt.2024.1472051

**Published:** 2024-10-25

**Authors:** Mads Bering Christiansen, Ahmad Rafsanjani, Jonas Jørgensen

**Affiliations:** SDU Soft Robotics, SDU Biorobotics, Mærsk Mc-Kinney Møller Institute, University of Southern Denmark, Odense, Denmark

**Keywords:** generative artificial intelligence, soft robotics, robot design, design aesthetics, biomorphism

## Abstract

Artificial Intelligence (AI) has rapidly become a widespread design aid through the recent proliferation of generative AI tools. In this work we use generative AI to explore soft robotics designs, specifically Soft Biomorphism, an aesthetic design paradigm emphasizing the inherent biomorphic qualities of soft robots to leverage them as affordances for interactions with humans. The work comprises two experiments aimed at uncovering how generative AI can articulate and expand the design space of soft biomorphic robotics using text-to-image (TTI) and image-to-image (ITI) generation techniques. Through TTI generation, Experiment 1 uncovered alternative interpretations of soft biomorphism, emphasizing the novel incorporation of, e.g., fur, which adds a new dimension to the material aesthetics of soft robotics. In Experiment 2, TTI and ITI generation were combined and a category of hybrid techno-organic robot designs discovered, which combined rigid and pliable materials. The work thus demonstrates in practice the specific ways in which AI image generation can contribute towards expanding the design space of soft robotics.

## Introduction

In recent years, artificial intelligence (AI) technology has become ubiquitous in a wide range of applications, revolutionizing how we tackle complex issues. Its impressive capabilities, increased accessibility, and cost-effectiveness have propelled its widespread adoption at drastic speeds. AI helps solve societal problems daily, within, e.g., healthcare (Gregory, 2023), agriculture (Dotson, 2020; Silverberg, 2023), and law enforcement (BBC News, 2023; Brewster, 2024). It has also attained a prominent role within aesthetic practice, e.g., in art and design (Choi, 2018; Reddy, 2022), architecture (Chaillou, 2020; Nayeri, 2023), fashion design (Y. K. Lee, 2022), and sound/music production (Savage, 2023). AI tools purportedly enable laypeople to carry out design-related tasks that were previously only possible for highly skilled professionals (Fowler and Lacey, 2023), and are also used by professionals to enhance human creativity and ideation (Figoli, 2022; Kim and Maher, 2023). It is, thus, no longer a question of *when* AI will change aesthetic, artistic, and designerly endeavors, but *how* these fields can benefit from incorporating AI tools in creative practice.

**FIGURE 1:**
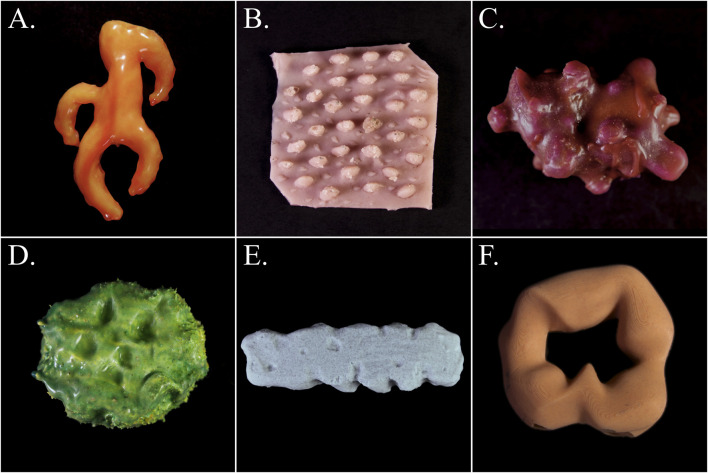
Examples of soft biomorphic robot prototypes (introduced in Christiansen et al. (2024b)): **(A)** “*Claw*”; **(B)** “*Rugged Surface*”; **(C)** “*Tuberous Form*”; **(D)** “*Green Oval*”; **(E)** “*Gray Tube*”; **(F)** “*Ring*”. Prototypes have been rescaled.

Motivated by the growing need for closer human-robot interactions in modern societies, the realm of robotics has witnessed the emergence of soft robotics as a novel paradigm in robot design, experiencing remarkable growth over the past two decades. Soft robotic systems are often biologically inspired and use compliant materials with mechanical properties similar to the tissue of soft biological organisms (Rus and Tolley, 2015). It has been argued that soft robots are more “lifelike” compared to hard-bodied robots (Laschi et al., 2016), and their suggested uses include assistance, rehabilitation, and collaborative work, as they offer safe close physical contact with humans (Arnold and Scheutz, 2017). The inherently different and organic aesthetic of soft robotics has also inspired artistic and designerly explorations of the technology (e.g., see Belling and Buzzo, 2021; Bering Christiansen et al., 2020; Bering Christiansen and Jørgensen, 2020; Bewley and Boer, 2018; Jørgensen, 2017). In prior work, we introduced the design paradigm of *Soft Biomorphism*, which aims to emphasize the inherent biomorphic aesthetics of soft robotics and leverage them as affordances for interaction with humans (Christiansen et al., 2024a; 2024b) (see Figure 1). Soft biomorphic robot designs accentuate visual, haptic, and kinetic similarities with natural soft organisms. The aim of soft biomorphism is to create lifelike yet unfamiliar robots to promote open-ended and negotiable human-robot relations. Simultaneously, the design aesthetic aligns with the principles of the *biophilia* hypothesis, which posits that humans possess an innate inclination to appreciate and connect with nature and other forms of life (Grinde and Patil, 2009; Kellert and Wilson, 1993; Wilson, 1984). By leveraging humans’ affinity for natural forms and behaviors, soft biomorphism aims to facilitate intuitive interactions with soft robots. Previous studies have investigated how generative AI, more specifically AI image generation, can blend and reimagine various components, creating innovative composites that surpass human imagination, offering valuable insights into object and product design (Broo, 2023; Gan et al., 2021; Hoggenmueller et al., 2023; Y. H. Lee and Chiu, 2023; Vartiainen and Tedre, 2023). Building on these insights, AI image generation may also uncover novel perspectives on soft biomorphism as a design aesthetic.

The work presented in this article aims to explore the application of generative AI to shed light on and extend soft biomorphism as a design aesthetic for soft robots. The investigation considers how soft biomorphism is depicted and understood by AI image generation software and how AI image generation can be used to articulate and extend this aesthetic. We further examine the meanings conveyed by the contents of the generated images and the visions of potential applications for soft biomorphic robots they propose.

In the context of generative AI, there are two main approaches for creating imagery: text-to-image (TTI) generation and image-to-image (ITI) generation. Our work is rooted in two experiments: firstly, we investigate how TTI generation interprets and extends text descriptions of the specific aesthetic of soft biomorphism; secondly, we combine TTI and ITI generation to explore what characterizes how soft biomorphic robots are pictured in the generated outputs. Lastly, we discuss the relationship between the generated images and the exposition of soft biomorphism given in our prior work (Christiansen et al., 2024b) and assess how the developed generative strategies can contribute to new interpretations of what soft biomorphism might entail. Through our exploration, we aim to offer insights that span the fields of design, visual studies, soft robotics, and applied AI.

To situate the work, we first provide an introduction to AI image generation that uses diffusion models and an overview of prior work that has utilized AI image generation for designing physical products and robots specifically. We continue with a brief introduction to the concept of biomorphism and the field of soft robotics followed by an exposition of their unification in the concept of soft biomorphism. After this, we present the methods used in the two experiments, followed by results and a discussion of each experiment, and finally a conclusion.

## Background: AI image synthesis with diffusion models

Generative models form a category within machine learning comprising models that can generate new data based on patterns detected in a set of training data. This technique is utilized in commercially available AI image generators such as Stable Diffusion (Stability AI, 2023), Midjourney (Midjourney, Inc, 2024), and DALL-E (OpenAI, 2024), which use diffusion models as an underlying framework for image generation. Diffusion models are probabilistic generative models, usually trained on immense sets of images with accompanying textual descriptions. Diffusion models operate through a two-stage process: first, a forward diffusion stage gradually introduces Gaussian noise into the input data; then, in the reverse diffusion stage, the model learns to reverse this process progressively, ultimately generating intelligible images as outputs (Croitoru et al., 2022; Rombach et al., 2022; Yang et al., 2023). This iterative process also involves denoising autoencoders that undergo training to accurately predict a denoised version of the input (Rombach et al., 2022), allowing diffusion models to efficiently produce even photorealistic images.

TTI generation combines probabilistic diffusion models with text-conditioned image generation techniques, synthesizing images from textual inputs known as “prompts” and “negative prompts” (Zhang et al., 2023). As textual descriptions accompany images in diffusion models, the model is taught the relationship between specific image contents and annotated text elements. Prompts then guide the outputs towards specific regions of the image space, while negative prompts instruct models to avoid certain types of outputs. In ITI generation, an existing image is used as input to generate, e.g., an output that improves the input image’s resolution, manipulates its colors, or an image that matches the content or visual style of the input image (Hugging Face, 2022). Many AI image generators allow users to combine visual and textual inputs, to guide the process to achieve desired outcomes like transferring visual styles of an input image and guiding the output content using textual prompts.

## Related work

### AI image generation within product design

With the recent advancements in AI image generation, there has been a surge in using this technology for exploration of the design space for physical products. In their experimental study, Y. H. Lee and Chiu (2023) investigated the effectiveness of using TTI generative AI tools, specifically Midjourney (Midjourney, Inc, 2024), compared to using the online image sharing site, Pinterest (Pinterest, Inc, 2024), for ideating industrial design products. The study aimed to investigate how these different tools influence the ideation process among industrial design students. Although the results did not show Midjourney to have superiority over Pinterest, Midjourney excelled in combining different styles and objects into unique compositions, suggesting its potential as a complementary tool alongside visual inspiration platforms online for ideating physical product designs. In the context of fashion design, Della Sciucca et al. (2022) presented a generative model capable of generating handbags through ITI generation. Their framework extracted line sketches from handbag images and transformed them into new handbag models, demonstrating the potential of generative models to inspire design creativity and aid in the development of physical products. Quan et al. (2023) demonstrated TTI generative AI’s capability to generate photorealistic images of complex product designs. They argued that AI image generation will enable designers to describe requirements in text rather than master traditional design skills. This transformative potential not only enhances cost-effectiveness but also encourages broader participation in the design process, as users from various backgrounds can easily communicate their design ideas without extensive training. Vartiainen and Tedre (2023) explored how TTI generation can be used in product design and craft education through workshops and discussions with craft teachers and educators. They reported that concerns arose related to the absence of physicality in AI-generated objects, potential copyright issues, and the risk of creativity being “black-boxed”. Their study, however, also highlighted positive aspects of TTI generative AI, including its ability to enhance ideation, generate novel objects, and offer new perspectives that trigger valuable reflections, even on physically impossible designs.

### AI image generation of robot designs

While a variety of generative AI techniques, such as the utilization of large language models (Stella et al., 2023), have been used to design robots, only a few studies have explored the use of AI image generation for designing robots. Investigating TTI generation for designing robots, Broo (2023) explored its potential to bring novel perspectives to robot design. They emphasized challenges in controlling the output alongside its ability to surprise with innovative design choices, such as material composition and overall form. Their work underscores TTI generation’s ability to inspire reflection on form and modes of locomotion, offering potentially valuable insights for physical robot design. Hoggenmueller et al. (2023) explored the transformative role of TTI generation in challenging design fixation and reshaping human-robot interaction (HRI) imaginaries. Over four weeks, participants used TTI generative AI tools in individual weekly design exercises and shared results digitally with the other participants. The study underscores positive experiences with using generative AI, its utility in visualizing diverse robot contexts, exposing existing HRI biases, and fostering imagination to arrive at novel designs. To generate novel social robot designs that align with customers’ preferences for appearance, Gan et al. (2021) trained a deep convolutional generative adversarial network on images of existing social robots. Their study showcased the efficacy of AI in aiding design professionals to ideate and refine social robot designs aligned with customer preferences, emphasizing the tool’s role as a supplement to rather than a replacement of robot designers in design processes.

In summary, prior research has examined the challenges and potentials of utilizing AI image generation for ideation in product design and robot design. Nevertheless, there remains a notable gap in the exploration of specific design aesthetics for physical objects through AI image generation. This study offers new insights into how generative AI can articulate and extend the soft biomorphic aesthetic.

### Biomorphism and soft robotics

**FIGURE 2:**
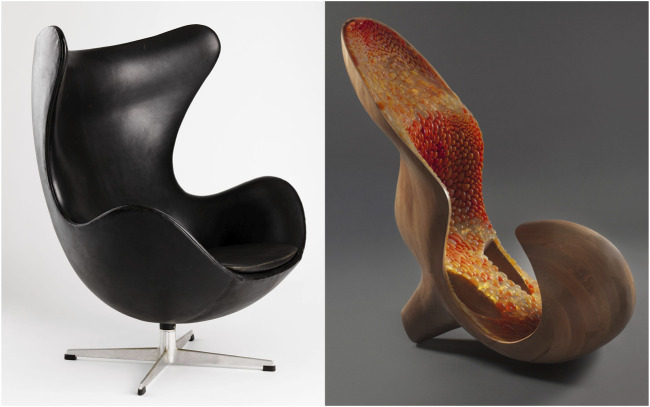
Examples of biomorphic furniture design. Left: Arne Jacobsen’s The Egg, 1958 (Photo: Nasjonalmuseet/Bjørgli, Annar. Available under a Creative Commons CC-BY 4.0 license at: https://www.nasjonalmuseet.no/en/collection/object/OK-1989-0185). Right: Gemini, 2014. Designed by Neri Oxman in collaboration with Prof. W. Craig Carter (MIT Department of Materials Science and Engineering) (Oxman et al., 2014).

Biomorphism, derived from the Greek words *bíos* (“life” or “living”) and *morphḗ* (“form”) (Agkathidis, 2017), is used in relation to art, architecture, and design practices to describe a preference for or use of organic, curvilinear, or lifelike forms (Botar, 2016) reminiscent of those found in natural organisms (Crowther and Wünsche, 2012). It denotes a biologically inspired, yet abstracted representation of aspects of the natural world (Wünsche, 2012) rather than faithful reproductions (Crowther and Wünsche, 2012). As a concept, biomorphism originates in late 19th-century anthropology (Haddon, 1895). An interest in the biomorphic formal language features prominently within art movements including Art Nouveau, Modernist abstraction, and Surrealism. This emergence coincided with novel discoveries of modern biology, e.g., making previously imperceptible organisms visible to the human eye (Juler, 2015). Examples of biomorphic aesthetics can be found in a wide range of works by renowned artists like Pablo Picasso, Hans Arp (Barr, 1936), and Henry Moore (Juler, 2015), architects such as Anthoni Gaudi (Imani, 2017), Frank Gehry, and Eero Saarinen (Barison, 2021), as well as designers like Alvar Aalto (Sandoval and Andrés, 2021), Isamu Noguchi (Gore, 2018), and Arne Jacobsen (The Art Story, 2024) (see Figure 2).

**FIGURE 3:**
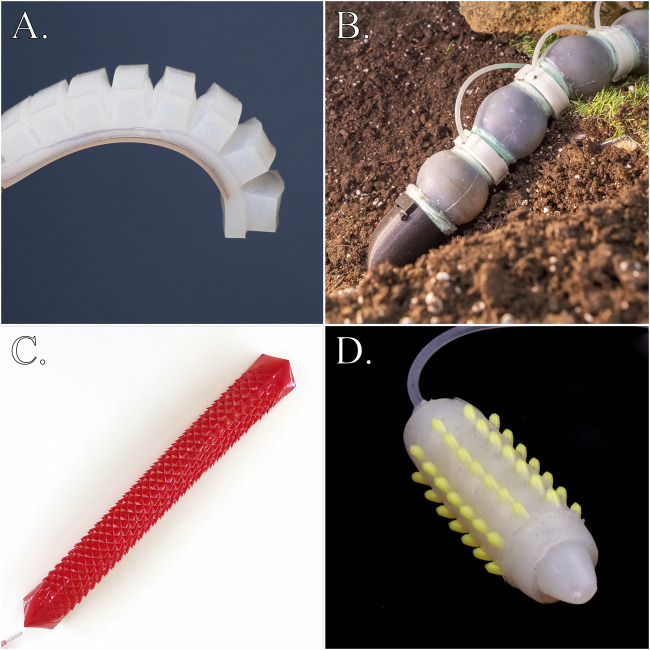
Examples of pneumatic soft robot designs: **(A)** soft silicone robot (Photo: UC San Diego Jacobs School of Engineering/David Baillot. Available under a Creative Commons CC-BY 2.0 license at: https://flic.kr/p/2eYwQy1. Image has been cropped); **(B)**. Earthworm-inspired modular soft robot by Das et al. (2023); **(C)**. Kirigami-skinned soft crawler robot by Rafsanjani et al. (2018); **(D)**. Earthworm-inspired soft skin crawling robot by Tirado et al. (2024).

Soft robots are autonomous machines currently being researched and developed that are primarily constructed from compliant, deformable materials, with mechanical properties similar to those of soft biological materials (Rus and Tolley, 2015) (see Figure 3). Using their compliance, soft robots can effectively adapt to uneven surfaces and complex objects, while demonstrating physical robustness by withstanding external impacts without sustaining damage, thus ensuring stability and safe operation around humans compared to hard-bodied robots (Laschi et al., 2017). Other major technical benefits include the possibility to base robot designs on fully or partially soft natural organisms, whose bodies have been shaped by evolution to diligently achieve certain mechanical tasks with ease. But also increased energy efficiency and improved safety in physical interactions with humans, by means of passive compliance. Soft robots have also been argued to possess an inherently lifelike appearance that enhances their potential for interaction with humans (Majidi, 2014).

In prior work, we introduced the concept of *soft biomorphism* as an alternate design paradigm for creating soft robots that centers on increasing their intrinsically organic aesthetic (Christiansen et al., 2024a; 2024b). This design paradigm has been explored in various physical soft robot designs (Christiansen et al., 2023; 2024b). The motivation for proposing soft biomorphism as a design principle is to create soft robots that appear lifelike while remaining unfamiliar, fostering more open-ended and adaptable interactions between humans and robots that are not modelled on specific interactions with, e.g., animals or pets. Previous work has suggested that robots with designs and behaviors open to interpretation may foster longer-term relationships between humans and robots by enabling more varied and engaging communication over time (Sandry, 2015). Designing robots after animal models can showcase a robot’s restricted behavioral, cognitive, or perceptual capabilities through its appearance (Löffler et al., 2020), aiding in setting suitable social expectations about a robot (Fong et al., 2003). However, basing robot designs on familiar animals has also been criticized as being unethical (Sparrow, 2002) and may create unrealistic expectations about a robot’s capabilities (Löffler et al., 2020). Consequently, it has been proposed that robot designs should be derived from unfamiliar animals to prevent misconceptions about a robot’s abilities (Breazeal, 2004). By abstracting nature rather than replicating specific animals or organisms, soft biomorphic robots can convey an impression of the robot as a responsive interaction partner through visual, haptic, or kinetic similarities with natural organisms in general. Furthermore, biomorphic robot designs can activate our natural inclination towards nature and other lifeforms (Grinde and Patil, 2009; Kellert and Wilson, 1993; Wilson, 1984) making the interaction more engaging and appealing. Although the biophilia hypothesis has later been challenged for lacking solid evidence (Joye and De Block, 2011), recent research highlights biophilic responses to nature exposure (see, e.g., Gaekwad et al., 2023; Schiebel et al., 2022), even when mediated through technology (Chan and Huang, 2024; Jung et al., 2023; Sabinson and Green, 2021). This suggests that while humans are increasingly accustomed to technology and artificial environments, bio-inspired design, such as soft biomorphic robot designs, may still offer benefits in technological contexts.

Soft biomorphism is thus used as a concept to characterize soft robot designs that accentuate visual, haptic, and kinetic resemblances with soft natural organisms. These robots may feature vibrant polychromatic colors and asymmetrical, bulbous forms and rugged surfaces. Softness in soft biomorphism implies material deformability, e.g., through the use of silicone materials, which also replicates the tactile sensations of touching soft-bodied organisms (Christiansen et al., 2024b). Instances of soft biomorphism range from abstract reinterpretations to naturalistic representation of partial elements, potentially blending traits from multiple organisms in one design. In essence, soft biomorphism entails the creation of organic appearing soft robot designs through the combination, variation, and hybridization of forms, tactility, and movements found in nature. These designs have potential applications in areas including care, entertainment, companionship, therapy, as well as social and personal robotics contexts (see Christiansen et al., 2024b).

## Methods

**FIGURE 4:**
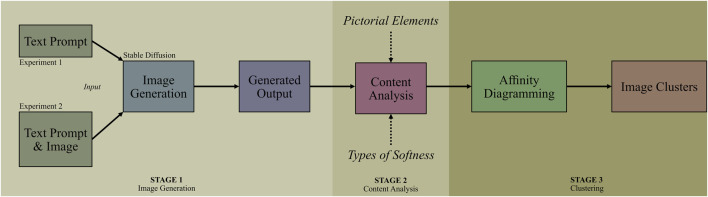
Overview of the visual data processing workflow. The process starts with image generation using Stable Diffusion based on either a text prompt (Experiment 1) or a combination of text prompt and image input (Experiment 2). The generated outputs undergo content analysis focusing on two variables: pictorial elements and types of softness. Finally, the images are grouped through Affinity Diagramming to form clusters based on visual characteristics and accompanying tags.

The methodology used in this work follows three overall stages (see Figure 4). First, image generation was performed, resulting in two sets of images. Second, content analysis was conducted to systematically map and quantify key visual elements and types of softness depicted in the generated images. Finally, through Affinity Diagramming, we clustered the images based on their visual characteristics and accompanying tags to identify overarching themes and design traits.

### Stable Diffusion configuration

We generated the images in *Experiment 1* (see Experiment 1: TTI generative AI) and *Experiment 2* (see Experiment 2: combining TTI and ITI generative AI) using Stable Diffusion (Stability AI, 2023), running locally on an Apple MacBook Pro (13″, M1, 2020, 16 GB RAM) *via* the AUTOMATIC1111 (2023) user interface. We selected Stable Diffusion for its configurable parameters (see Table 1). For a more detailed description of these parameters, see Supplementary Material 1. In each experiment, Stable Diffusion was set to generate 64 outputs.

**TABLE 1:** Overview of configurable parameters used in Stable Diffusion to generate the outputs in Experiment 1 and 2.

Parameter	Description	Used in experiment	Name/Value
*Checkpoint*	Pre-trained model used for image generation	1 and 2	v1-5-pruned-emaonly checkpoint
*Seed*	Starting point for image noise generation	1 and 2	−1
*Sampling Method*	Algorithm for denoising from random noise	1 and 2	Euler Ancestral
*Sampling Steps*	Number of steps in the generation process	1 and 2	50
*CFG Scale*	Controls how closely the image matches the text prompt	1 and 2	9
*Denoising Strength*	Controls similarity to input image	2	0.75

### Analysis of images

Content analyses were conducted on the two resulting image sets from each experiment to map and quantify their contents. Content analysis is a systematic method for categorizing the contents of images based on distinct variables with a discrete set of values to differentiate the characteristics of a delimited set (Bell, 2011). Initially, the first author and the corresponding author analyzed a sample set of 32 images, after which each suggested potential variables of interest for the content analysis. Through discussion, they mutually agreed on two categories for the analyses of both image sets: *pictorial elements* and *types of softness*. These categories were used in the subsequent analysis. The first author analyzed the remaining images, with input from the corresponding author in case of doubt about the categorization to ensure consistency and accuracy. Manual image annotation by tagging was used to establish relevant values for each variable. Manual image annotation is commonly used within computer science to assign textual metadata (e.g., captions, and descriptive text) to images, to facilitate description of the semantic contents of large image sets (Adnan et al., 2020; Yan et al., 2007). This methodology was chosen due to the exploratory nature of the study as it enabled us to inductively establish suitable values for each variable derived from the data. The images were annotated one by one, with observed values added continually under each variable.

The first variable, pictorial elements, was chosen to shed light on and allow for comparison between the motifs of each of the two image sets. Under pictorial elements we chose to only include the objects and materials visible in the image. The rationale for combining objects and materials in the same category was that some elements present in the generated images were not recognizable as specific objects, but their material could still be identified. This approach to tagging ensured that we could capture all relevant visual information. For this variable, each image received between 1–10 tags describing its characteristics, resulting in 32 different pictorial elements in *Experiment 1* and 25 different pictorial elements in *Experiment 2* (the full list of pictorial elements can be found in Supplementary Materials 3, 4). The decision to not use a fixed number of tags and have no lower limit on tags was made to encompass images with varying degrees of details and number of elements and to only include tags of high relevance to describing the imagery. To annotate pictorial elements, we used nouns and noun phrases (e.g., “hard plastic”, and “plush toy”) to exhaustively tag all clearly visible object(s) and material(s) contained in each of the generated images. For each image the different pictorial elements were established one by one until all elements of the image had been tagged.

To understand how our configuration of Stable Diffusion interpreted the meaning of “soft”, in soft robotics and soft biomorphism, we included the depicted types of softness as the second variable. We specifically chose to include this variable to acknowledge that “soft” has multiple meanings and explore which of these were activated in the generated image sets. E.g., “soft” can encompass objects “*having [a] curved or rounded outline*” (Merriam-Webster, 2024), objects that are “*changing [their] shape[s] when pressed*” (Cambridge Dictionary, 2024), and objects that are “*smooth and pleasant to touch*” (Britannica Dictionary, 2024). To annotate the depicted objects’ types of softness, we used nouns and noun phrases. Through the tagging process, the following four values for types of softness were established:

• Organic form: Softness expressed through the overall shape or form of an object, encompassing curvilinear and rounded outlines.
• Pliable material: Softness attributable to the inclusion of pliable or deformable materials and matter, e.g., skin, rubber, fabric, plants, and fruits.
• Surface smoothness: Softness conveyed through a smooth surface, where the absence of rugged surface textures contributes to a visually smooth and soft appearance.
• Fur: Softness related to the inclusion of dense hairs on a surface.

For images that contained more than one type of softness, all values were amended as tags.

Following content analyses, to identify groups of similar images, for each experiment we categorized the images and their accompanying tag annotations through the method of *Affinity Diagramming*. This method is commonly used in design processes for organizing large amounts of unstructured qualitative data into cohesive categories (Hartson and Pyla, 2012; Holtzblatt and Beyer, 2015). Remaining images that did not fit into major categories were consolidated into a category labelled “other” and excluded from further analysis, to focus the analysis on overarching themes and patterns.

## Experiment 1: TTI generative AI

The goal of the first experiment was to examine how generative AI comprehends and translates text descriptions of soft robots with soft biomorphic aesthetics into visual representations. We aimed to explore how the selected generative AI method understands, interprets, and extends text descriptions of this aesthetic and to leverage TTI image generation as a tool to arrive at alternative or more nuanced understandings of what a soft biomorphic design aesthetic might entail.

### Procedure

We experimented with prompts and negative prompts (see Supplementary Material 2) using keywords from our text description of soft biomorphism (see Christiansen et al., 2024b and Biomorphism and soft robotics). Drawing from comprehensive guides on authoring Stable Diffusion prompts (Ma, 2023; Monge, 2023; Stable Diffusion Art, 2023), we included prompts and negative prompts to shape the visual style of the generated images. E.g., we included the prompts “DSLR” and “RAW” and the negative prompts “painting”, and “drawing” to make the outputs appear like photographs depicting physically existing objects. Through our iterative experimentation that spanned five rounds, each generating 64 images, our aim was to generate images portraying physical robot designs and objects imbued with biomorphic traits. Following the initial round dominated by white plastic humanoid robots, we introduced prompts to emphasize biomorphic and organic forms. However, in the subsequent round, this mainly resulted in landscape images, deviating from our objectives. Thus, in the third round, we adjusted prompts to robot design with “biologically inspired and biomorphic visual appearance and form”. Additionally, we added “photo studio setting” as a prompt to steer away from the landscape images and achieve a detailed object depiction with a shallow depth of field and lighting as in studio photography. These changes steered us away from landscape images but mainly yielded monochrome toy-like objects. Despite removing prompts for “natural colors” to allow for a wider range of colors in the objects, there was not a noticeable difference in the objects’ colors in the fourth round of images. In the fifth round, we added prompts accentuating organic materials and introduced prompts for “vivid organic polychromatic coloring and nuances”, while removing the negative prompt for “saturated colors” to enhance color variety in the objects. Ultimately, we settled on the prompts from the fifth round, as they produced a diverse range of object types with varied colors and materials.

The following prompts and negative prompts were used to generate the image set in *Experiment 1*:

**Prompt:** ((full-body image of a soft robot with a biologically inspired and biomorphic visual appearance, form and surface texture, set in a photo studio:1.3)), (photo studio setting:1.5), (soft robot made from organic looking material:2), ((pliable materials)), pliable, (biomorphic form)), (robot surface has vivid organic polychromatic coloring and nuances:1.3), (biomorphic robot), (organic form), (organic surface), (((soft natural organism))), asymmetrical, bulbous, rugged, arciform, sweeping, annular, undulating and irregular contours, photography, RAW, DSLR, high resolution, HiRes, High quality.

**Negative Prompt:** (plastic), ((metal)), painting, drawing, cartoon, rendering, 3D, computer graphics, saturated, blurry, ((low resolution)), LoRes, (bad quality).

### Results

The total time required to generate the image set in *Experiment 1* was approximately 102 min. The 64 TTI-generated images with annotations and categorizations can be seen in Supplementary Material 3.

#### Content analysis

**FIGURE 5:**
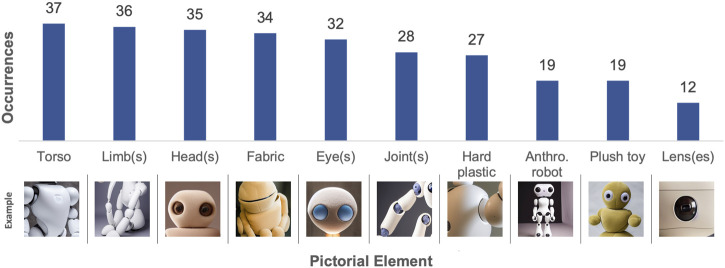
Results from content analysis of the TTI-generated images in Experiment 1 - The ten most frequently occurring pictorial elements. Example images have been cropped.

**FIGURE 6:**
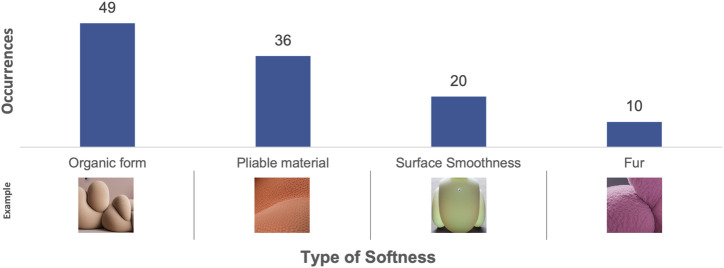
Results from content analysis of the TTI-generated images in Experiment 1 - The types of softness depicted. Example images have been cropped.

The content analysis revealed a total of 32 different pictorial elements in *Experiment 1* (see Supplementary Material 3). The ten most frequently occurring pictorial elements are listed in Figure 5 and the types of softness depicted are listed in Figure 6.

As seen in Figure 5, body parts were the most pervasive pictorial elements, with torsos, limbs, heads, and eyes being the most frequently occurring. Additionally, synthetic materials such as hard plastic were prevalent, and the TTI output images tended to use artificial materials for robot construction.

#### Categorization

**FIGURE 7:**
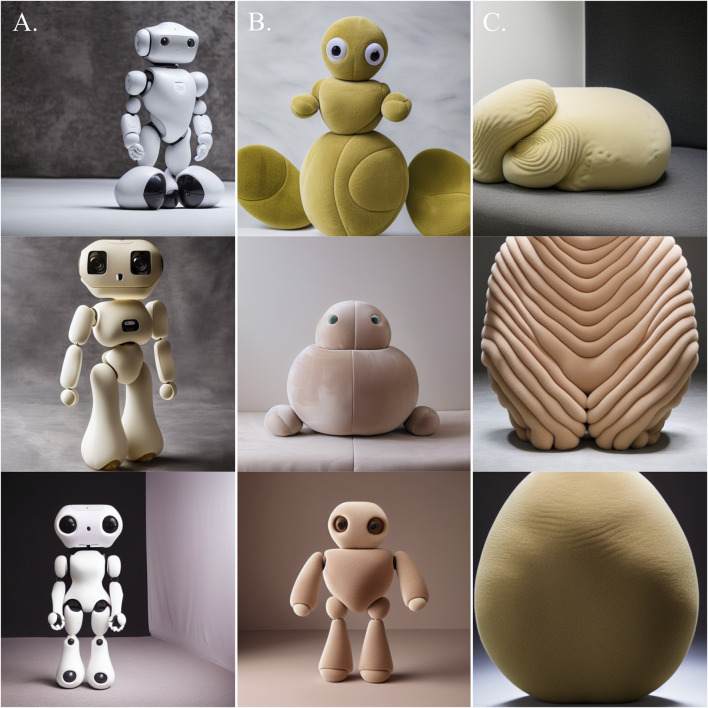
Examples of the three general categories of objects generated through TTI generative AI - from left to right: **(A)** Anthropomorphic Robots; **(B)** Plush Toys; **(C)** Biomorphic Sculptures.

Using Affinity Diagramming, we established three overarching categories of objects in the TTI-generated images (see Figure 7):

• Anthropomorphic Robots
• Plush Toys
• Biomorphic Sculptures

The first category, Anthropomorphic Robots (n = 19), features anthropomorphic body parts including limbs, torsos, and heads (see Figure 7A). They have rounded outer casings made from sleek and glossy hard plastic, along with visible joints between the body parts. Additionally, some designs feature caricatured wide eyes and small mouths. Softness is mainly depicted through the plastic’s even surface smoothness (n = 18) and curvilinear, sweeping, organic forms (n = 17).

The second category, Plush Toys (n = 19), features objects with visual similarities to plush toys (see Figure 7B). These objects feature rounded forms and simplified versions of body structures seen in animals, e.g., limbs, torsos, heads, and eyes. They are primarily composed of physically soft and compliant materials such as fabrics (n = 15), fur (n = 2), and skin (n = 2). The types of softness represented in the objects are predominantly organic overall forms (n = 19) and pliable materials (n = 17).

The third category contains what we dub Biomorphic Sculptures (n = 12), referring to objects resembling highly abstract organic entities rather than specific animals (see Figure 7C). These objects feature irregular, curvilinear, and bulging forms yet are devoid of clear resemblances with specific animals. In terms of materials, most of the objects are covered in smooth yet rumpled and uneven fur or short hair (n = 8). Thus, softness is included via organic forms (n = 12), fur (n = 8), and pliable materials (n = 4).

### Discussion

#### Social robotics prevail over soft robotics

A commonality between the outputs of *Experiment 1* is missing or only vague resemblance to existing soft robotics designs, which are often composed of simple geometrical shapes assembled to mimic the overall morphology of a distinct animal or animal body part (Jørgensen, 2019) (see Figure 3). Notably absent are elements resembling pneumatic soft actuators and robots characterized by the use of opaque, semitranslucent silicone with sleek surfaces (see, e.g., Rus and Tolley, 2015; Walker et al., 2020) (see Figure 3).

Designs in the Anthropomorphic Robot category instead resemble existing social robot designs like SoftBank Robotics’ *NAO, Pepper,* and UBTECH’s *Lynx* and use similar color palettes for the humanoid bodies. Some designs in the Plush Toys category also feature caricatured anthropomorphic bodies but differ by also incorporating materials like fabric and skin. This depiction of robots as human replicas is a typical feature of social robots (Dunstan and Hoffman, 2023). The robots in the category of Anthropomorphic Robots thus appear as if designed to also mimic human behavior and take part in human-like social interaction. Furthermore, their use of hard plastic suggests that they are not designed for physical contact but instead for performing menial tasks or engaging in verbal communication. Consequently, the designs in the Anthropomorphic Robot category align more with established notions of humanoid social robots rather than showcasing the adaptive and tactile capabilities typically associated with soft robotics.

#### Likeness to abstract art and soft robotic artworks

Contrary to the Anthropomorphic Robot category, the fur-covered objects within the category of Biomorphic Sculptures offer a novel take on how soft robots – let alone social robots – might look. These objects display ambiguous, abstract, organic forms, yet elude clear resemblance to distinct organisms. Their distended shapes make them appear more like sculptures than robot morphologies. The simple, irregular, and curvilinear forms bear similarities to abstract sculptural works by 20th century artists, including Hans Arp and Henry Moore, which dwell in visual ambiguity and openness of form. While examples of soft robots embodying similar abstract and biomorphic organic forms as those in the Biomorphic Sculptures exist, these are interestingly not found within technical soft robotics research. Instead, such examples figure within the realm of artistic explorations of soft robotics, e.g., Paula Gaetano Adi’s spherical-shaped robotic latex sculpture, *Alexitimia* (2006/2007), and the protruding, biomorphic inflations of the soft robotic silicone tiles in Jonas Jørgensen and Maja Smrekar’s robotic installation, *!brute_force: Soft Resilience* (2022). By avoiding representing familiar shapes, the Biomorphic Sculptures category of robots appears to invite open-ended exploration of their interaction possibilities. Their likeness to sculptures and soft robotic artworks underscores the potential of creative uses of generative AI to supplement artistic explorations of robotics to inform, enrich, and extend the design space of soft robotics.

#### Fur – another type of softness

Interestingly, most designs in the category of Biomorphic Sculptures are covered in hair and in that sense depart from the notion of softness as material deformability prevalent within soft robotics research (Rus and Tolley, 2015). Body hair is a product of evolution (Lesnik-Oberstein, 2011) that carries strong cultural meaning, as it has historically been associated with wild beasts and physical strength (Hallpike, 1969). In the context of robotics, faux fur has been used for pet-like robots (Flagg et al., 2012; Goris et al., 2011; Müller et al., 2015; Schellin et al., 2020; Singh and Young, 2013; Stiehl et al., 2006). While most commercial social robots with fur mimic specific animals, e.g., PARO Robotics’ *PARO* seal, Fujitu’s *Teddy Bear*, and Joy for All’s *Companion Pet* series, other fur-covered robot designs diverge from this trend. E.g., MIT Media Lab’s *Tega* (Westlund et al., 2016) and Vanguard Industries’ *Moflin.* Both resemble imaginary creatures and use fur to evoke lifelike qualities without emulating existing animals. Indeed, prior research shows that adding fur to a robot can also positively impact the emotional states of users (Mitsui et al., 2001) and reduce perceptions of it as scary (Schellin et al., 2020). While deviating from the conventional concept of softness within soft robotics, fur’s association with natural entities and positive effects on human-robot interaction makes it promising to include fur as a different kind of softness within soft biomorphism. This addition can enhance not only the tactile and emotional aspects but also the visual resemblance to soft natural organisms, enriching the overall interaction with soft biomorphic robots.

## Experiment 2: combining TTI and ITI generative AI

In preliminary work, we explored soft biomorphism through ITI generation by using photos of soft biomorphic prototypes (see Christiansen et al., 2024a; Christiansen et al., 2024b) as image sources. These tests, however, did not yield favorable results as the output images tended to closely mirror the shape and colors of the prototypes pictured in the input images and omit reference to robotics. Consequently, we chose to combine TTI and ITI generation for the second experiment to explore potential synergy between the two input modalities for the stated purpose of the current work, and to investigate how the results of the combined approach differ from those obtained with TTI generation.

### Procedure

**FIGURE 8:**
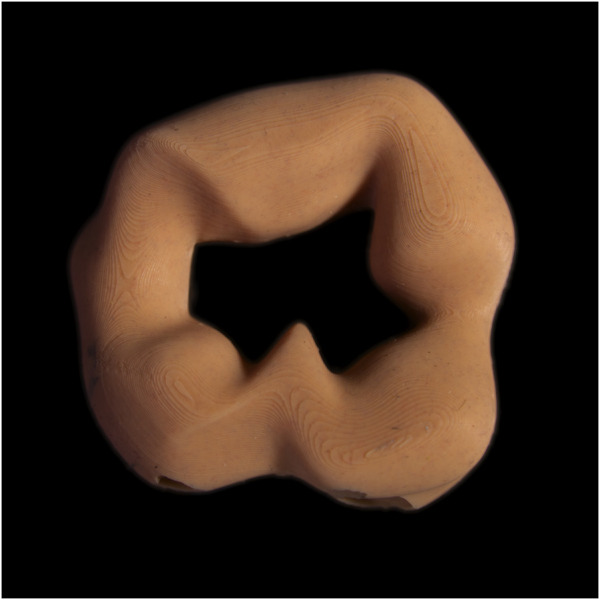
Photograph of the soft biomorphic robot prototype “*Ring*” used as input image in Experiment 2.

As visual input we used a photograph of a previously designed soft biomorphic robot prototype, “*Ring*” (see Figure 8), introduced in Christiansen et al. (2024b). We conducted initial trials by combining the input image with the prompts used in *Experiment 1*. These preliminary trials resulted in outputs that were very similar to those from *Experiment 1*, particularly featuring plush-like objects with fabric as a dominant material. This replication suggested that the existing prompts were insufficient for extending the biomorphic aesthetic beyond previous findings with ITI generation. To allow for a fuller exploration of soft biomorphism as a design aesthetic for soft robots, we modified the prompts to broaden the scope of objects and materials allowed in the generated outputs.

The following prompts and negative prompts were used in combination with the image input (Figure 8) to generate the image set in *Experiment 2*:

**Prompt:** ((full-body image of a soft-bodied robot with a biologically inspired and biomorphic visual appearance, form and surface texture:1.3)), (robot made from organic looking material:2), (biomorphic form)), (robot surface has vivid organic polychromatic coloring and nuances:1.3), (biomorphic robot), (organic form), (organic surface), (((soft natural organism))), asymmetrical, bulbous, rugged, arciform, sweeping, annular, undulating and irregular contours, photography, RAW, DSLR, high resolution, HiRes, High quality.

**Negative Prompt:** (plastic), ((metal)), painting, drawing, cartoon, rendering, 3D, computer graphics, saturated, blurry, ((low resolution)), LoRes, (bad quality).

### Results

The generation of the image set in *Experiment 2* took approximately 81 min. The 64 combined ITI and TTI-generated images and their annotations and categorizations can be found in Supplementary Material 4.

#### Content analysis

**FIGURE 9:**
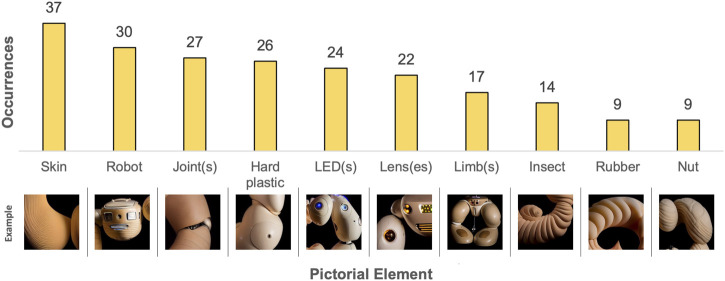
Results from content analysis of the combined ITI and TTI-generated images in Experiment 2 - The ten most frequently occurring pictorial elements. Example images have been cropped.

**FIGURE 10:**
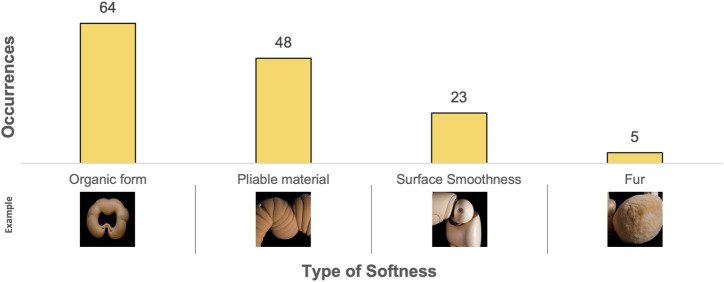
Results from content analysis of the combined ITI and TTI-generated images in Experiment 2 - The types of softness depicted. Example images have been cropped.

The content analysis revealed a total of 25 different pictorial elements (see Supplementary Material 4). The ten most frequently occurring pictorial elements are listed in Figure 9 and the different types of softness depicted are listed in Figure 10.

In *Experiment 2*, a diverse array of organic categories emerged, prominently featuring skin and insect-related elements among the most frequent pictorial elements. Through this shift towards organic materials the outcomes deviate from reliance on synthetic materials observed in *Experiment 1* (see Content analysis). It is important to note that the prompts were modified going from *Experiment 1* to *Experiment 2* to broaden the range of generated outputs. While these modifications were necessary to achieve our research objectives, they also affect the comparability of results between the two experiments. Therefore, any comparisons made are and should be considered in relation to this difference.

Comparing the ten most frequently occurring pictorial elements occurring in *Experiment 2* (see Figure 9) to those of *Experiment 1* (see Figure 5) reveals a small overlap, including hard plastic, joints, and limbs. This suggests a consistent bias towards these elements in the generated imagery, regardless of whether TTI or combined ITI and TTI generation is used and regardless of prompt modifications. However, *Experiment 2* introduces unique categories not observed in *Experiment 1*’s most frequently occurring pictorial elements, including skin, LEDs, lenses, insects, rubber, and nuts. Particularly skin indicates a departure from *Experiment 1*’s synthetic materials, possibly influenced by the input image of soft biomorphic prototype “*Ring*” (see Figure 8) and the changed prompts. Furthermore, the overall forms of the generated objects in *Experiment 2* are all ring-shaped, suggesting that the image of “*Ring*” set certain limitations on the types of imagery that could be generated.

Interestingly, the rankings of types of softness depicted across both experiments (see Figures 6, 10) exhibit a consistent ratio between the different types. This consistency suggests a uniform pattern in the generative capabilities of the AI system, irrespective of the input modality and changes to the prompts.

#### Categorization

**FIGURE 11:**
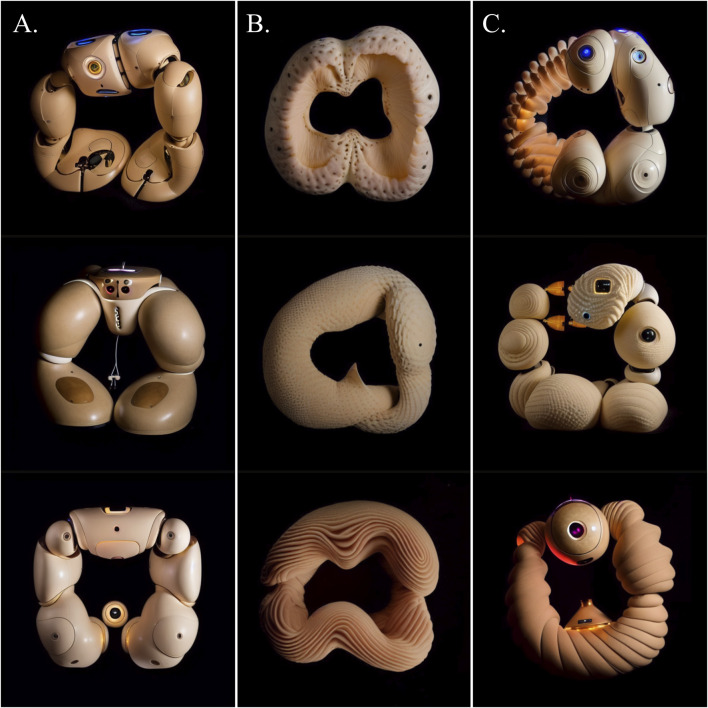
Examples of the three general categories of objects generated through combined TTI and ITI generative AI - from left to right: **(A)** Limbed Robots; **(B)** Biomorphic Entities; **(C)** Hybrid Techno-Organic Robots.

Through Affinity Diagramming, the generated outcomes of the TTI and ITI generation were categorized into three distinct categories of objects (see Figure 11):

• Limbed Robots
• Biomorphic Entities
• Hybrid Techno-Organic Robots

The first category, Limbed Robots (n = 11) contains hard plastic structures with limb-like appendages attached via joints to a central body part, resulting in symmetrical morphologies (see Figure 11A). These robots have eye-like lenses, predominantly placed in the smaller center parts of the robots. The most frequently occurring pictorial elements are hard plastic (n = 11), joints between body parts (n = 11), arm or leg-like limbs (n = 10), LEDs (n = 9), and lenses (n = 10). Softness is mainly portrayed through sleek surface smoothness (n = 11) and the morphologies’ rounded forms (n = 6).

Biomorphic Entities (n = 33), the second category, includes organic ring-shaped biomorphic entities principally with surfaces resembling skin (see Figure 11B). These biomorphic objects appear as abstract versions of natural soft organisms, with the most frequently occurring pictorial elements being skin (n = 24), nuts (n = 9), insect segments (n = 7), fingers (n = 6), and reptile segments (n = 4). The types of softness depicted primarily concern soft sinuous, flowing organic forms (n = 31) as well as inclusion of pliable materials (n = 26).

The third category, Hybrid Techno-Organic Robots (n = 16), contains ring-shaped objects that fuse features of natural soft tissue with rigid elements, including hard plastic, glass, and electronic components, resulting in complex morphologies that appear neither fully technological nor fully organic (see Figure 11C). Within this distinct “*techno-organic*” aesthetic, traits from both natural organisms and contemporary robotics technology appear to seamlessly coexist. The most frequently occurring pictorial elements are LEDs (n = 11), joints (n = 11), skin (n = 10), hard plastic (n = 9), and lenses (n = 8). Various types of softness are depicted simultaneously, particularly organic forms (n = 16), pliable materials (n = 13), and smooth surfaces (n = 10).

### Discussion

#### Novel types of biohybrid soft robot designs

The most surprising and novel designs to emerge in *Experiment 2* were in the category of Hybrid Techno-Organic Robots, which were made from a mix of living and non-living elements, as well as rigid and pliable materials. This category mirrors existing interests within soft robotics research not only in using rigid and pliable materials together within robot morphologies (Christiansen et al., 2023; Larsen et al., 2022; Stokes et al., 2014) but also to mix living and non-living materials (Park et al., 2016). The Hybrid Techno-Organic Robots mix mechanical robot parts made from hard plastic or metal with appendages seemingly made from organic materials, blending these clashing elements with different aesthetics and associations to form unique robot designs. One might be tempted to categorize them as either robotic technology with organic exteriors, or organisms enhanced with technological components, *cyborgs* integrating biological and technological elements through fusion of human and machine (Warwick, 2012). Combining organic and electronic elements like this is a recurrent trope within the cultural imaginary (Dixon, 2007). Within the strand of cybernetic robot artworks since the 1960s (Burnham, 1969), e.g., Thomas Shannon’s robotic sculpture *Squat* (1966) is an early notable example, which integrates a living plant with electronic components to create an “*organic/inorganic hybrid*” (Kac, 2001) and suggests the coexistence of nature and machines within a unified system. Kakoudaki (2014) notes similar ideas of blending technology with biology within popular culture, e.g., movie characters like Ash in “Alien*”* (Scott, 1979) and T-800 in “The Terminator” (Cameron, 1984) have skin-like surfaces and mechanical components integrated in android bodies. These characters represent dystopian visions of highly advanced societies, where natural-looking exteriors hide technology, aiding them in blending in. Presenting a novel design aesthetic for soft robotics technology, the Hybrid Techno-Organic Robots simultaneously carry such iconographic meanings, representing both harmonious and dystopian visions of merging two distinctive aesthetics in one morphology. Therefore, the fusion of organic and mechanical elements in the Hybrid Techno-Organic Robots prompts critical questions about their possible roles in interactions with humans. While organic features may evoke familiarity and comfort akin to natural organisms, they also blur the boundaries between living and non-living entities, introducing complexities into how we might perceive them, and the ethical stakes of pursuing such designs. This fusion of natural and mechanical elements allows for a reimagining of robot functionality and potential interactions, where the interplay between organic and inorganic materials opens up new possibilities for design and application.

#### Biological robots

The objects in the category Biomorphic Entities are thought-provoking as they challenge conventional notions of “robotics” by appearing as if crafted solely from biological materials. Particularly their inclusion of skin-like materials and reptile and insect segments results in an appearance of biological growth, starkly contrasting with the fabricated appearances of *Experiment 1*’s Anthropomorphic Robots and *Experiment 2*’s Limbed Robots, and partly the Hybrid Techno-Organic Robots, all featuring joints and electronic components. The Biomorphic Entities’ inclusion of organic forms and natural materials invite contemplation of the relationship between technology and biology, a subject currently under investigation in biohybrid and cyborganic systems (e.g., see Orive et al., 2020; Ricotti et al., 2017). A well-known example within the context of soft robotics is the creation of tiny Xenobot robots composed of frog embryo cells (Kriegman et al., 2020). This not only expands the possibilities for soft robotics but also challenges traditional notions of what constitutes a robot. The incorporation of biological materials in this category, thus, offers a glimpse into the potential future of soft biomorphic robots, showcasing potential biohybrid or cyborganic systems and the prospect of growing robots from living materials, that do not look like the robots we have now. The potential for growing rather than constructing robots also opens up innovative pathways in manufacturing and maintenance, where biological processes could be harnessed for self-repair and adaptation.

## Conclusion

This work has aimed to explore the utilization of generative AI to illuminate soft biomorphism as a design aesthetic for soft robots. Specifically, it sought to uncover and expand the formal characteristics and associated meanings of soft biomorphism as a design space through the application of AI image generation tools, namely, TTI generation and combined TTI and ITI generation.

The two experiments using AI image generation gave rise to new understandings of soft biomorphism and interpretations of what this design aesthetic might encompass. The AI-generated objects manifested diverse forms of biomorphism, ranging from subtle organic influences to pronounced biomorphic overall appearances. Notably, some output images featured coherent morphologies that combined elements with varying degrees of realism, i.e., with varying degrees of likeness to specific identifiable animal body parts. This represents a departure from our previous design strategy for creating soft biomorphic prototypes (Christiansen et al., 2024b) that aimed at attaining a consistent level of realism for all their parts, which lies between exact replication and total abstraction. This coexistence of inconsistencies in realism of elements could offer novel directions for future robot designs. The different parts of the robot’s body could be made to vary in their level of resemblance to the body parts of a specific animal, to conjure up or play down association between the robot and this animal. Thereby affordances for interaction established by means of people’s knowledge of the animal may be included alongside more abstract design elements that do not come with specific associations about behavior and interaction. Another important novel understanding of soft biomorphism emerged through the analysis of the materials included in the objects. Some of the AI-generated outputs incorporated, e.g., skin-like materials and fur, also extending our prior notion of soft biomorphism, while still maintaining affinity with natural organisms. Incorporating a wider range of different materials in soft biomorphic robots is worthy of further exploration in future soft biomorphic robot designs, as certain materials may serve to guide the intended interaction based on their cultural connotations and uses. Finally, the mix of different materials propose a novel composite understanding of softness, as entailing multiple dimensions (pliability, organic forms, surface smoothness, fur, *etc.*), which significantly expands the original definition of soft biomorphism provided in prior work. These designs thus also challenge the definition of softness used commonly used within soft robotics.

This exploration of soft biomorphism and soft robot aesthetics in AI-generated designs also highlights potential limitations of using generative AI in achieving truly novel designs. For instance, the Anthropomorphic Robots category exemplifies the biases inherent in the generative AI’s training data and societal assumptions about what characterizes a robot’s design. Similarly, this is evident in the category of Plush Toys in which pliable materials were predominantly linked to objects resembling plush toys, reinforcing preconceived associations between certain materials and designs. While the generative AI’s replication of existing robot and object designs may seem unsurprising, it raises important questions about the biases embedded within the training data and the generative process itself. Hoggenmueller et al. (2023) explains that the designer-AI co-creative process can serve as a platform for highlighting existing robot stereotypes and confronting these embedded biases. This raises the broader question about generative AI’s usefulness for exploring novel design aesthetics. As noted by Manovich (2024), AI models are trained by extracting patterns from data, eliminating outliers and unique details, and prioritizing the most frequently occurring associations, characteristics, and structures. This compression leads to the preservation of the “average” while uncommon traits and unique variations are often removed. Thus, while our work demonstrates how AI-generated imagery can inspire novel interpretations of soft biomorphism, it is essential to also recognize the inherent limitations in AI’s capacity to innovate beyond the existing patterns embedded in its training data. This tension highlights a key issue to take into account when using this tool for exploration of robot designs.

Our work demonstrates the usefulness and limitations of AI image generation as a tool to explore soft robot design aesthetics. The AI-generated images afforded diverse interpretations of soft biomorphism and soft robot morphologies, suggesting novel combinations and new avenues for soft robotics designs.

## Limitations

The presented work is subject to some limitations. Firstly, the methodology described in this article does not take into account whether it is technically possible to build the robots depicted in the generated images. While AI image generation is a valuable tool for exploring aesthetic robot designs, it does not consider their technical feasibility. Another generative system could be developed in future work, informed by existing soft robotics parts and modules, ensuring that the designs are realizable. However, the present study focuses on exploring soft robot design aesthetics and soft biomorphism, rather than practical implementation.

Similarly, focusing predominantly on aesthetics could come at the cost of mechanical functionality of the robot. Aesthetic choices could theoretically impact functionality, e.g., using materials like fur or sticky surfaces might interfere with the robot’s ability to perform specific tasks or move smoothly. However, in several cases, aesthetic elements can be incorporated without affecting the mechanical function at all (e.g., a robot’s color, pattern, or surface texture). In contrast to our focus on aesthetics, evolutionary robotics employs AI technology to develop robot designs purely from a functional standpoint (see, e.g., Bongard, 2013; Doncieux et al., 2015; Husbands, 2010). As these approaches differ, one emphasizing aesthetics and the other functionality, the integration of AI tools into robotics design currently requires the expertise of human robot designers to bridge the gap. Our work fits within this context, using AI image generation as a tool for robot designers, that may offer aesthetic expressions that can inspire and inform the design process, but not replace the need for human expertise in their construction.

Another limitation regards that the content analyses were carried out by two of the authors. While striving to be objective and systematic in our analysis, involving independent reviewers or additional analysts may have provided more diverse perspectives and reduced potential bias.

Finally, a general limitation of using the soft biomorphic aesthetic for soft robots is that some designs could sometimes be perceived as off-putting, weird, or alien, due to appearing too organic or through odd juxtapositions of body parts or natural elements. However, this potential limitation could also become an opportunity for intentional design choices. Robot designers could incorporate unappealing traits that can be activated or deactivated to evoke discomfort or unease, to end the interaction with the robot or to convey caution.

## Data Availability

The original contributions presented in this work are included in the Supplementary Material. Further inquiries can be directed to the corresponding author.

## References

[B1] Adnan M. M. Mohd Rahim M. S. Al-Jawaheri K. Ali M. H. Waheed S. R. Radie A. H. (2020). “A survey and analysis on image annotation,” in 2020 3rd International Conference on Engineering Technology and Its Applications (IICETA), Najaf, Iraq, 06-07 September 2020, 203–208. 10.1109/iiceta50496.2020.9318911

[B2] Agkathidis A. (2017). Biomorphic structures: architecture inspired by nature (form + technique). London, United Kingdom: Laurence King Publishing.

[B3] Arnold T. Scheutz M. (2017). The tactile ethics of soft robotics: designing wisely for human–robot interaction. Soft Robot. 4 (2), 81–87. 10.1089/soro.2017.0032 29182090

[B4] AUTOMATIC1111 (2023). Stable diffusion web UI [Python]. Available at: https://github.com/AUTOMATIC1111/stable-diffusion-webui.

[B5] Barison M. (2021). Beyond the organic paradigm biomorphic digital architecture. Aesthetica Prepr. 117. Available at: https://mimesisjournals.com/ojs/index.php/aesthetica-preprint/article/view/1668.

[B6] Barr A. H. (1936). Cubism and abstract art. Mus. Mod. Art. New York, NY: The Museum of Modern Art 1936. Available at: https://assets.moma.org/documents/moma_catalogue_2748_300086869.pdf.

[B7] BBC News (2023). AI cameras catch 297 drivers in three days in Cornwall. BBC News. Available at: https://www.bbc.com/news/uk-england-cornwall-66508840.

[B8] Bell P. (2011). Content analysis of visual images. The handbook of visual analysis. London, United Kingdom: SAGE Publications Ltd. 10–34. 10.4135/9780857020062

[B9] Belling A.-S. Buzzo D. (2021). The rhythm of the robot: a prolegomenon to posthuman somaesthetics. Proc. Fifteenth Int. Conf. Tangible, Embed. Embodied Interact., 1–6. 10.1145/3430524.3442470

[B10] Bering Christiansen M. Beloff L. Jørgensen J. Belling A.-S. E. (2020). Soft robotics and posthuman entities. J. Artistic Res. 22. 10.22501/jar.549014

[B11] Bering Christiansen M. Jørgensen J. (2020). Augmenting soft robotics with sound. Companion 2020 ACM/IEEE Int. Conf. Human-Robot Interact., 133–135. 10.1145/3371382.3378328

[B12] Bewley H. Boer L. (2018). *Designing blo-nut*: design principles, choreography and otherness in an expressive social robot. Proc. 2018 Des. Interact. Syst. Conf., 1069–1080. 10.1145/3196709.3196817

[B13] Bongard J. C. (2013). Evolutionary robotics. Commun. ACM 56 (8), 74–83. 10.1145/2493883

[B14] Botar O. (2016). “Biomorphism,” in Routledge encyclopedia of modernism. 1st ed. (Oxfordshire, United Kingdom: Routledge). 10.4324/9781135000356-REM770-1

[B15] Breazeal C. (2004). Designing sociable robots. MIT Press.

[B16] Brewster T. (2024). This AI watches millions of cars daily and tells cops if you’re driving like A criminal. Forbes. Available at: https://www.forbes.com/sites/thomasbrewster/2023/07/17/license-plate-reader-ai-criminal/ (Accessed July 19, 2023).

[B17] Britannica Dictionary (2024). Soft. Definition and meaning. Available at: https://www.britannica.com/dictionary/soft (Accessed November 13, 2023).

[B18] Broo D. G. (2023). How generative AI helped me imagine a better robot: it didn’t give me schematics, but it did boost my creativity. IEEE Spectr. 60 (11), 44–50. 10.1109/MSPEC.2023.10309274

[B19] Burnham J. (1969). Real time systems. New York, NY: Artforum. Available at: https://www.artforum.com/features/real-time-systems-210752/.

[B20] Cambridge Dictionary (2024). Soft—definition. Available at: https://dictionary.cambridge.org/dictionary/english/soft (Accessed November 13, 2023).

[B116] Cameron J. (Director) (1984). The terminator [Film]. Orion Pictures.

[B21] Chaillou S. (2020). “ArchiGAN: artificial intelligence x architecture,” in Architectural intelligence: selected papers from the 1st international conference on computational design and robotic fabrication (CDRF 2019). Editors Yuan P. F. Xie M. Leach N. Yao J. Wang X. (Springer Nature), 117–127. 10.1007/978-981-15-6568-7_8

[B22] Chan H. Huang Y. (2024). “Research on the benefits of biophilia effects in virtual environments,” in Virtual, augmented and mixed reality. Editors Chen J. Y. C. Fragomeni G. (Switzerland: Springer Nature), 16–28. 10.1007/978-3-031-61044-8_2

[B23] Choi S. K. (2018). Guess, check and fix: a phenomenology of improvisation in ‘neural’ painting. Digit. Creat. 29 (1), 96–114. 10.1080/14626268.2018.1423995

[B24] Christiansen M. B. Asawalertsak N. Do C. D. Nantareekurn W. Rafsanjani A. Manoonpong P. (2023). “BioMORF: a soft robotic skin to increase biomorphism and enable nonverbal communication. 2023,” in 2023 32nd IEEE International Conference on Robot and Human Interactive Communication, Busan, Korea, Republic of, 28-31 August 2023 (New York, NY: IEEE), 370–377. 10.1109/RO-MAN57019.2023.10309420

[B25] Christiansen M. B. Rafsanjani A. Jørgensen J. (2024a). Ex silico: soft biomorphs. able. Available at: https://able-journal.org/en/ex-silico.

[B26] Christiansen M. B. Rafsanjani A. Jørgensen J. (2024b). “It brings the good vibes”: exploring biomorphic aesthetics in the design of soft personal robots. Int. J. Soc. Robotics 16, 835–855. 10.1007/s12369-023-01037-6

[B27] Croitoru F.-A. Hondru V. Ionescu R. T. Shah M. (2022). Diffusion models in vision: a survey. IEEE Trans. Pattern Analysis Mach. Intell. 45, 10850–10869. 10.1109/TPAMI.2023.3261988 37030794

[B28] Crowther P. Wünsche I. (2012). “Introduction,” in Meanings of abstract art. Editors Crowther P. Wünsche I. (Oxfordshire, United Kingdom: Routledge).

[B29] Das R. Babu S. P. M. Visentin F. Palagi S. Mazzolai B. (2023). An earthworm-like modular soft robot for locomotion in multi-terrain environments. Sci. Rep. 13 (1), 1571. 10.1038/s41598-023-28873-w 36709355 PMC9884293

[B30] Della Sciucca L. Balloni E. Mameli M. Frontoni E. Zingaretti P. Paolanti M. (2022). “StyleTrendGAN: a deep learning generative framework for fashion bag generation,” in Image analysis and processing. ICIAP 2022 workshops. Editors Mazzeo P. L. Frontoni E. Sclaroff S. Distante C. (Springer International Publishing), 191–202. 10.1007/978-3-031-13324-4_17

[B31] Dixon S. (2007). Digital performance: a history of new Media in theater, dance, performance art, and installation. MIT Press. Available at: http://ebookcentral.proquest.com/lib/sdub/detail.action?docID=3338680.

[B32] Doncieux S. Bredeche N. Mouret J.-B. Eiben A. E. (2015). Evolutionary robotics: what, why, and where to. Front. Robotics AI 2. 10.3389/frobt.2015.00004

[B33] Dotson K. (2020). Blue River harnesses AI to help farmers combat invasive weeds. Palo Alto, CA: SiliconANGLE. Available at: https://siliconangle.com/2020/08/06/blue-river-technology-harnesses-ai-help-farmers-combat-invasive-weeds/.

[B34] Dunstan B. J. Hoffman G. (2023). “Social robot morphology: cultural histories of robot design,” in Cultural robotics: social robots and their emergent cultural ecologies. Editors Dunstan B. J. Koh J. T. K. V. Turnbull Tillman D. Brown S. A. (Springer International Publishing), 13–34. 10.1007/978-3-031-28138-9_2

[B35] Figoli F. (2022). AI in design idea development: a workshop on creativity and human-AI collaboration. DRS2022 Bilbao. 10.21606/drs.2022.414

[B36] Flagg A. Tam D. MacLean K. Flagg R. (2012). Conductive fur sensing for a gesture-aware furry robot. 2012 IEEE Haptics Symp. (HAPTICS), 99–104. 10.1109/HAPTIC.2012.6183776

[B37] Fong T. Nourbakhsh I. Dautenhahn K. (2003). A survey of socially interactive robots. Robotics Aut. Syst. 42 (3), 143–166. 10.1016/S0921-8890(02)00372-X

[B38] Fowler G. A. Lacey E. (2023). Anyone can Photoshop now, thanks to AI’s latest leap. Wash. Post. Available at: https://www.washingtonpost.com/technology/2023/06/16/ai-photoshop-generative-fill-review/.

[B39] Gaekwad J. S. Sal Moslehian A. Roös P. B. (2023). A meta-analysis of physiological stress responses to natural environments: biophilia and Stress Recovery Theory perspectives. J. Environ. Psychol. 90, 102085. 10.1016/j.jenvp.2023.102085

[B40] Gan Y. Ji Y. Jiang S. Liu X. Feng Z. Li Y. (2021). Integrating aesthetic and emotional preferences in social robot design: an affective design approach with Kansei Engineering and Deep Convolutional Generative Adversarial Network. Int. J. Industrial Ergonomics 83, 103128. 10.1016/j.ergon.2021.103128

[B41] Gore H. (2018). Art/work: J.B. Blunk, Isamu Noguchi and the intersection of sculpture and skilled labor. J. Mod. Craft 11 (1), 17–25. 10.1080/17496772.2018.1440808

[B42] Goris K. Saldien J. Vanderborght B. Lefeber D. (2011). Mechanical design of the huggable robot probo. Int. J. Humanoid Robotics 08 (03), 481–511. 10.1142/S0219843611002563

[B43] Gregory A. (2023). New artificial intelligence tool can accurately identify cancer. Guard. Available at: https://www.theguardian.com/society/2023/apr/30/artificial-intelligence-tool-identify-cancer-ai.

[B44] Grinde B. Patil G. G. (2009). Biophilia: does visual contact with nature impact on health and well-being? Int. J. Environ. Res. Public Health 6 (9), 2332–2343. 10.3390/ijerph6092332 19826546 PMC2760412

[B45] Haddon A. C. (1895). Evolution in art: as illustrated by the life-histories of designs. Limited.

[B46] Hallpike C. R. (1969). Social hair. Man 4 (2), 256–264. 10.2307/2799572

[B47] Hartson R. Pyla P. S. (2012). The UX book: process and guidelines for ensuring a quality user experience. Elsevier.

[B48] Hoggenmueller M. Lupetti M. L. van der Maden W. Grace K. (2023). Creative AI for HRI design explorations. Companion 2023 ACM/IEEE Int. Conf. Human-Robot Interact., 40–50. 10.1145/3568294.3580035

[B49] Holtzblatt K. Beyer H. (2015). Contextual design: evolved. Springer International Publishing. 10.1007/978-3-031-02207-4

[B50] Hugging Face (2022). What is Image-to-Image? Available at: https://huggingface.co/tasks/image-to-image.

[B51] Husbands P. (2010). “Evolutionary robotics,” in Encyclopedia of machine learning. Editors Sammut C. Webb G. I. (Springer US), 373–382. 10.1007/978-0-387-30164-8_285

[B52] Imani M. (2017). Bio-inspired design approach analysis: a case study of antoni Gaudi and santiago calatrava. Int. J. Archit. Environ. Eng. 11 (8), 1156–1162. 10.5281/zenodo.1132046

[B53] Jørgensen J. (2017) “Prolegomena for a transdisciplinary investigation into the materialities of soft systems,” in ISEA 2017 manizales: bio-creation and peace: proceedings of the 23rd international symposium on electronic art. Colombia: University of Caldas, Manizales, 153–160.

[B54] Jørgensen J. (2019). Constructing soft robot aesthetics: art, sensation, and materiality in practice. Copenhagen, Denmark: IT University of Copenhagen. PhD thesis.

[B55] Joye Y. De Block A. (2011). Nature and I are two’: a critical examination of the biophilia hypothesis. Environ. Values 20 (2), 189–215. 10.3197/096327111X12997574391724

[B56] Juler E. (2015). Grown but not made: British modernist sculpture and the new biology. 1st edition. Manchester, United Kingdom: Manchester University Press.

[B57] Jung D. Kim D. I. Kim N. (2023). Bringing nature into hospital architecture: machine learning-based EEG analysis of the biophilia effect in virtual reality. J. Environ. Psychol. 89, 102033. 10.1016/j.jenvp.2023.102033

[B58] Kac E. (2001). The origin and development of robotic art. Convergence 7 (1), 76–86. 10.1177/135485650100700108

[B59] Kakoudaki D. (2014). Anatomy of a robot: literature, cinema, and the cultural work of artificial people. Rutgers University Press. 10.36019/9780813562179

[B60] Kellert S. R. Wilson E. O. (1993). The biophilia hypothesis. Washington, DC: Island Press.

[B61] Kim J. Maher M. L. (2023). The effect of AI-based inspiration on human design ideation. Int. J. Des. Creativity Innovation 0 (0), 81–98. 10.1080/21650349.2023.2167124

[B62] Kriegman S. Blackiston D. Levin M. Bongard J. (2020). A scalable pipeline for designing reconfigurable organisms. Proc. Natl. Acad. Sci. 117 (4), 1853–1859. 10.1073/pnas.1910837117 31932426 PMC6994979

[B63] Larsen B. Manoonpong P. Jørgensen J. (2022). WISARD: weight informing soft artificial robotic dermis. IEEE 5th Int. Conf. Soft Robotics (RoboSoft), 1–8. 10.1109/RoboSoft54090.2022.9762174

[B64] Laschi C. Iida F. Rossiter J. Cianchetti M. Margheri L. (2017). “Introduction,” in Soft robotics: trends, applications and challenges. Editors Laschi C. Rossiter J. Iida F. Cianchetti M. Margheri L. (Springer International Publishing), 1–4. 10.1007/978-3-319-46460-2_1

[B65] Laschi C. Mazzolai B. Cianchetti M. (2016). Soft robotics: technologies and systems pushing the boundaries of robot abilities. Sci. Robotics 1 (1), eaah3690. 10.1126/scirobotics.aah3690 33157856

[B66] Lee Y. H. Chiu C. Y. (2023). “The impact of AI text-to-image generator on product styling design,” in Human interface and the management of information. Editors Mori H. Asahi Y. (Switzerland: Springer Nature), 502–515. 10.1007/978-3-031-35132-7_38

[B67] Lee Y. K. (2022). How complex systems get engaged in fashion design creation: using artificial intelligence. Think. Ski. Creativity 46, 101137. 10.1016/j.tsc.2022.101137

[B68] Lesnik-Oberstein K. (2011). The last taboo: women and body hair. Manchester, United Kingdom: Manchester University Press. 10.7228/manchester/9780719075001.001.0001

[B69] Löffler D. Dörrenbächer J. Hassenzahl M. (2020). “The uncanny valley effect in zoomorphic robots: the U-shaped relation between animal likeness and likeability,” in Proceedings of the 2020 ACM/IEEE international conference on human-robot interaction (New York, NY: Association for Computing Machinery), 261–270. 10.1145/3319502.3374788

[B70] Ma Y. (2023). Stable diffusion prompt guide for beginners. San Diego, CA: AiTuts. Available at: https://aituts.com/stable-diffusion-prompts/.

[B71] Majidi C. (2014). Soft robotics: a perspective—current trends and prospects for the future. Soft Robot. 1 (1), 5–11. 10.1089/soro.2013.0001

[B72] Manovich L. (2024). “Separate and reassemble: generative AI and Media history,” in Artificial aesthetics: generative AI. Editors Manovich L. Arielli E. (Art and Visual Media). Available at: https://manovich.net/index.php/projects/artificial-aesthetics.

[B73] Merriam-Webster (2024). Soft. Available at: https://www.merriam-webster.com/dictionary/soft (Accessed November 13, 2023).

[B74] Midjourney, Inc (2024). Midjourney . Available at: https://www.midjourney.com/home (Accessed October 1, 2024).

[B75] Mitsui T. Shibata T. Wada K. Touda A. Tanie K. (2001). Psychophysiological effects by interaction with mental commit robot. Proc. 2001 IEEE/RSJ Int. Conf. Intelligent Robots Syst. Expand. Soc. Role Robotics Next Millenn. (Cat. No.01CH37180) 2, 1189–1194. 10.1109/IROS.2001.976330

[B76] Monge J. C. (2023). How to write the best stable diffusion prompts in 2023. Miami, FL: Hackr.Io. Available at: https://hackr.io/blog/stable-diffusion-prompts.

[B77] Müller S. Schröter C. Gross H.-M. (2015). “Smart Fur tactile sensor for a socially assistive mobile robot,” in Intelligent robotics and applications. Editors Liu H. Kubota N. Zhu X. Dillmann R. Zhou D. (Springer International Publishing), 49–60. 10.1007/978-3-319-22876-1_5

[B78] Nayeri F. (2023). How A.I. Is helping architects change workplace design. N. Y. Times. Available at: https://www.nytimes.com/2023/06/15/business/workplace-design-zhai-ai.html.

[B79] OpenAI (2024). DALL·E 3. Available at: https://openai.com/dall-e-3 (Accessed March 11, 2024).

[B80] Orive G. Taebnia N. Dolatshahi-Pirouz A. (2020). A new era for cyborg science is emerging: the promise of cyborganic beings. Adv. Healthc. Mater. 9 (1), 1901023. 10.1002/adhm.201901023 31778037

[B114] Oxman N. Dikovsky D. Belocon B. Carter W. C. (2014). Gemini: Engaging Experiential and Feature Scales Through Multimaterial Digital Design and Hybrid Additive–Subtractive Fabrication. 3D Printing and Additive Manufacturing 1 (3), 108–114. 10.1089/3dp.2014.1505

[B81] Park S.-J. Gazzola M. Park K. S. Park S. Di Santo V. Blevins E. L. (2016). Phototactic guidance of a tissue-engineered soft-robotic ray. Science 353 (6295), 158–162. 10.1126/science.aaf4292 27387948 PMC5526330

[B82] Pinterest, Inc (2024). Pinterest. Available at: https://www.pinterest.com/ (Accessed March 11, 2024).

[B83] Quan H. Li S. Zeng C. Wei H. Hu J. (2023). Big data and AI-driven product design: a survey. Appl. Sci. 13 (16), 9433. Article 16. 10.3390/app13169433

[B84] Rafsanjani A. Zhang Y. Liu B. Rubinstein S. M. Bertoldi K. (2018). Kirigami skins make a simple soft actuator crawl. Sci. Robotics 3 (15), eaar7555. 10.1126/scirobotics.aar7555 33141681

[B85] Reddy A. (2022). Artificial everyday creativity: creative leaps with AI through critical making. Digit. Creat. 33 (4), 295–313. 10.1080/14626268.2022.2138452

[B86] Ricotti L. Trimmer B. Feinberg A. W. Raman R. Parker K. K. Bashir R. (2017). Biohybrid actuators for robotics: a review of devices actuated by living cells. Sci. Robotics 2 (12), eaaq0495. 10.1126/scirobotics.aaq0495 33157905

[B87] Rombach R. Blattmann A. Lorenz D. Esser P. Ommer B. (2022). “High-resolution image synthesis with latent diffusion models,” in 2022 IEEE/CVF conference on computer vision and pattern recognition (CVPR), 10674–10685. 10.1109/CVPR52688.2022.01042

[B88] Rus D. Tolley M. T. (2015). Design, fabrication and control of soft robots. Nature 521 (7553), 467–475. 10.1038/nature14543 26017446

[B89] Sabinson E. B. Green K. E. (2021). “How do we feel? User perceptions of a soft robot surface for regulating human emotion in confined living spaces,” in 2021 30th IEEE International Conference on Robot and Human Interactive Communication, Vancouver, BC, Canada, 08-12 August 2021 (New York, NY: IEEE), 1153–1158. 10.1109/RO-MAN50785.2021.9515499

[B90] Sandoval J. M. J. Andrés J. R. (2021). Alvar Aalto: El vínculo con Jean Arp y la estructura interna de los objetos biomórficos. EGA Rev. Expresión Gráfica Arquit. 26 (42), 142. 10.4995/ega.2021.13919

[B91] Sandry E. (2015). Re-Evaluating the form and communication of social robots. Int. J. Soc. Robotics 7 (3), 335–346. 10.1007/s12369-014-0278-3

[B92] Savage M. (2023). AI-generated Drake and the Weeknd song goes viral. BBC News. Available at: https://www.bbc.com/news/entertainment-arts-65298834.

[B93] Schellin H. Oberley T. Patterson K. Kim B. Haring K. S. Tossell C. C. (2020). Man’s new best friend? Strengthening human-robot dog bonding by enhancing the doglikeness of sony’s aibo. 2020 Syst. Inf. Eng. Des. Symposium (SIEDS), 1–6. 10.1109/SIEDS49339.2020.9106587

[B94] Schiebel T. Gallinat J. Kühn S. (2022). Testing the Biophilia theory: automatic approach tendencies towards nature. J. Environ. Psychol. 79, 101725. 10.1016/j.jenvp.2021.101725

[B115] Scott R. (Director) (1979). Alien [Film]. 20th Century Fox.

[B95] Silverberg D. (2023). Lasers, drones and AI: the future of weeding. BBC News. Available at: https://www.bbc.com/news/business-64742513.

[B96] Singh A. Young J. E. (2013). “A dog tail for utility robots: exploring affective properties of tail movement,” in Human-computer interaction – interact 2013. Editors Kotzé P. Marsden G. Lindgaard G. Wesson J. Winckler M. (Springer), 403–419. 10.1007/978-3-642-40480-1_27

[B97] Sparrow R. (2002). The March of the robot dogs. Ethics Inf. Technol. 4 (4), 305–318. 10.1023/A:1021386708994

[B98] Stability AI (2023). Generative models by stability AI. Available at: https://github.com/Stability-AI/generative-models.

[B99] Stable Diffusion Art (2023). Stable Diffusion prompt: a definitive guide. Available at: https://stable-diffusion-art.com/prompt-guide/.

[B100] Stella F. Della Santina C. Hughes J. (2023). How can LLMs transform the robotic design process? Nat. Mach. Intell. 5 (6), 561–564. 10.1038/s42256-023-00669-7

[B101] Stiehl W. D. Breazeal C. Han K.-H. Lieberman J. Lalla L. Maymin A. (2006). The huggable: a therapeutic robotic companion for relational, affective touch. ACM SIGGRAPH 2006 Emerg. Technol. 10.1145/1179133.1179149

[B102] Stokes A. A. Shepherd R. F. Morin S. A. Ilievski F. Whitesides G. M. (2014). A hybrid combining hard and soft robots. Soft Robot. 1 (1), 70–74. 10.1089/soro.2013.0002

[B103] The Art Story (2024). Biomorphism: movement overview. Art Story. Available at: https://www.theartstory.org/movement/biomorphism/ (Accessed September 19, 2023).

[B104] Tirado J. Do C. D. Moisson de Vaux J. Jørgensen J. Rafsanjani A. (2024). Earthworm-inspired soft skin crawling robot. Adv. Sci. 11 (23), 2400012. 10.1002/advs.202400012 PMC11187925 38622890

[B105] Vartiainen H. Tedre M. (2023). Using artificial intelligence in craft education: crafting with text-to-image generative models. Digit. Creat. 34 (1), 1–21. 10.1080/14626268.2023.2174557

[B106] Walker J. Zidek T. Harbel C. Yoon S. Strickland F. S. Kumar S. (2020). Soft robotics: a review of recent developments of pneumatic soft actuators. Actuators 9 (1), 3. 10.3390/act9010003

[B107] Warwick K. (2012). “Cyborgs,” in Encyclopedia of applied ethics. Editor Chadwick R. Second Edition (Academic Press), 699–704. 10.1016/B978-0-12-373932-2.00028-4

[B108] Westlund J. K. Lee J. J. Plummer L. Faridi F. Gray J. Berlin M. (2016). Tega: a social robot. 11th ACM/IEEE Int. Conf. Human-Robot Interact. (HRI), 561. 10.1109/HRI.2016.7451856

[B109] Wilson E. O. (1984). Biophilia. Harvard University Press.

[B110] Wünsche I. (2012). “Life into art: nature philosophy, the life sciences, and abstract art,” in Meanings of abstract art. Editors Crowther P. Wünsche I. (Oxfordshire, United Kingdom: Routledge).

[B111] Yan R. Natsev A. Campbell M. (2007). An efficient manual image annotation approach based on tagging and browsing. Workshop Multimedia Inf. Retr. Many Faces Multimedia Semant., 13–20. 10.1145/1290067.1290071

[B112] Yang L. Zhang Z. Song Y. Hong S. Xu R. Zhao Y. (2023). Diffusion models: a comprehensive Survey of Methods and applications. ACM Comput. Surv. 56 (4), 105:1–105:39. 10.1145/3626235

[B113] Zhang C. Zhang C. Zhang M. Kweon I. S. (2023). *Text-to-image diffusion Models in generative AI: a survey* (arXiv:2303.07909). arXiv. Available at: http://arxiv.org/abs/2303.07909 . 10.48550/arXiv.2303.07909

