# Supplementary material for: Nature redux: interrogating biomorphism and soft robot aesthetics through generative AI
Source: Front Robot AI. 2024 Oct 25;11:1472051. doi: 10.3389/frobt.2024.1472051 (PMC11543949; doi:10.3389/frobt.2024.1472051)

Supplementary Material 2

Prompt Experiments:

64 TTI generated images - 1st round

SETTINGS

Prompt: ((full-body image of a soft robot)), (soft-bodied robot), ((Soft materials)), pliable, pliant, (biomorphism), (((biomorphic robot design))), (organism), (((organic))), (soft natural organism), asymetrical, bulbous, rugged, arciform, sweeping, annular, undulating and irregular contours, photography, RAW, Nikon DSLR, high resolution, HiRes, High quality, (natural colors)

Negative prompt: (plastic), ((metal)), painting, drawing, cartoon, rendering, 3D, computer graphics, out of proportions, (saturated colors) , saturated, blurry, ((low resolution)), LoRes, (bad quality)

Steps: 50, Sampler: Euler a, CFG scale: 9, Seed: -1, Size: 512x512, Model hash: 6ce0161689, Model: v1-5-pruned-emaonly, Version: v1.6.0

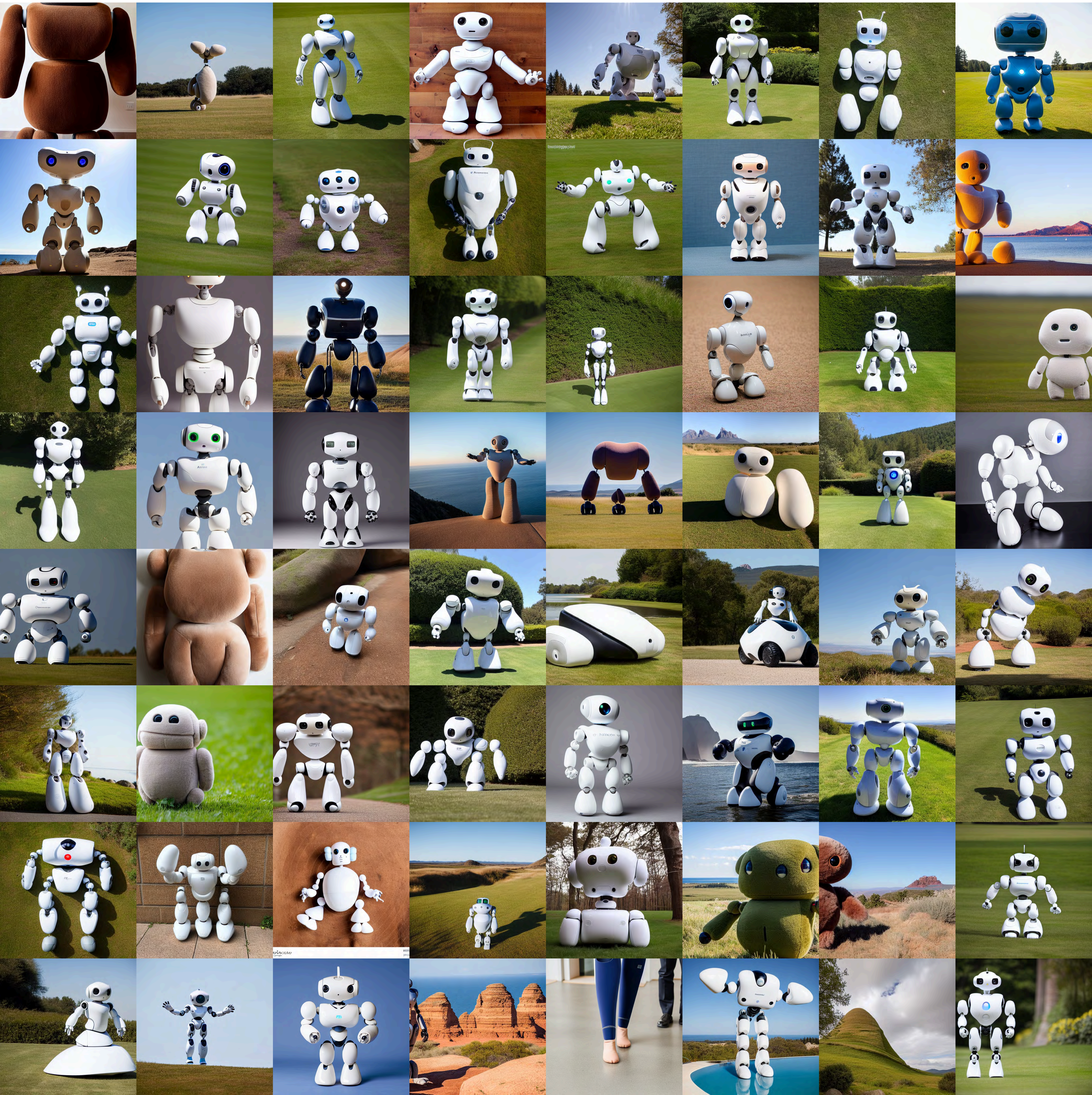

Prompt Experiments:
64 TTI generated images - 2nd round

SETTINGS

Prompt: ((full-body image of a soft robot)), (soft robot), (soft-bodied robot), ((Soft materials)), pliable, pliant, ((biomorphism)), (biomorphic form)), (biomorphic robot), (organism), ((organic form)), (((soft natural organism))), asymetrical, bulbous, rugged, arciform, sweeping, annular, undulating and irregular contours, photography, RAW, DSLR, high resolution, HiRes, High quality, (natural colors)

Negative prompt: (plastic), ((metal)), painting, drawing, cartoon, rendering, 3D, computer graphics, out of proportions, (saturated colors) , saturated, blurry, ((low resolution)), LoRes, (bad quality)

Steps: 50, Sampler: Euler a, CFG scale: 9, Seed: -1, Size: 512x512, Model hash: 6ce0161689, Model: v1-5-pruned-emaonly, Version: v1.6.0

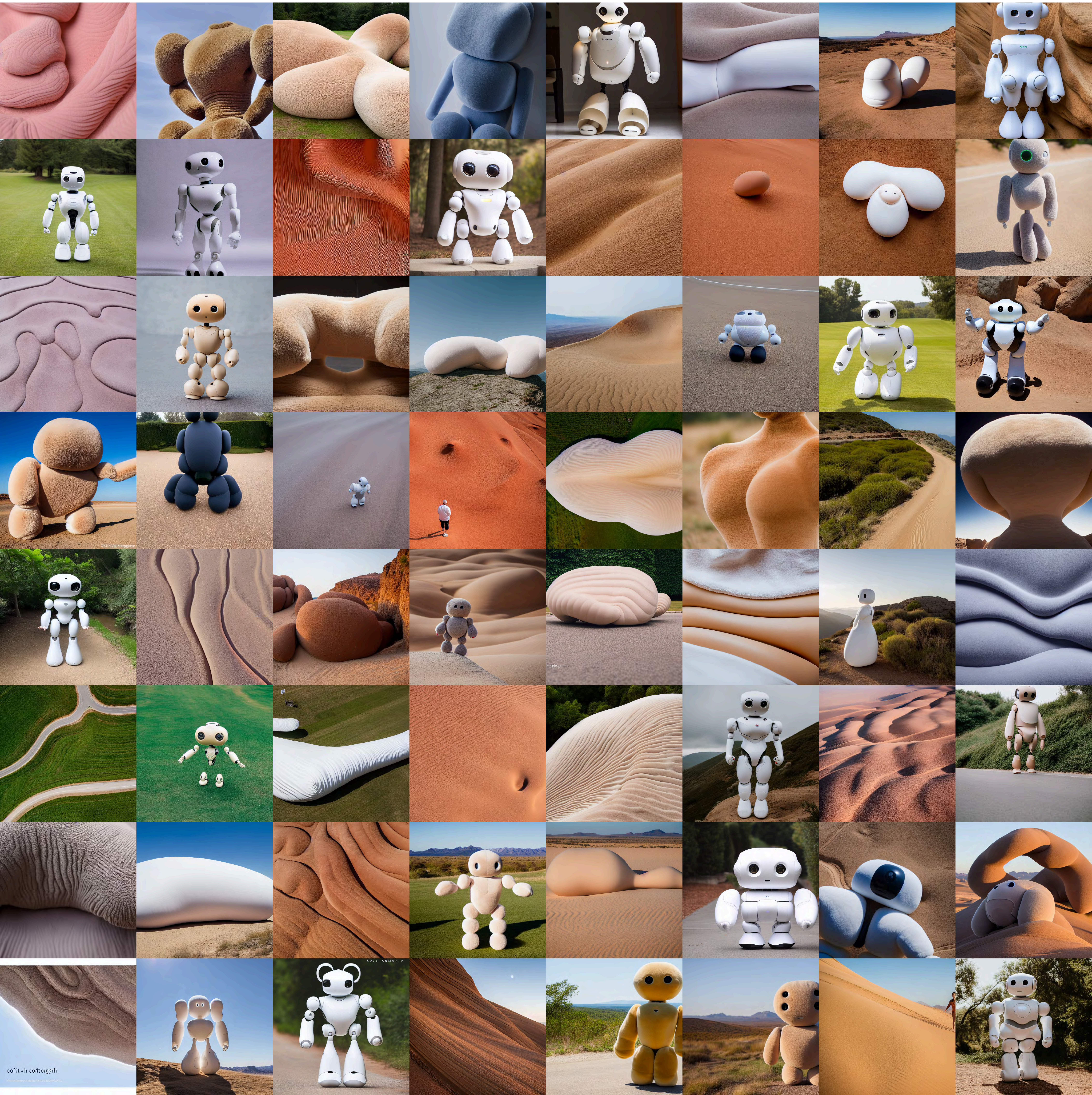

Prompt Experiments:
64 TTI generated images - 3rd round

SETTINGS

Prompt: ((full-body image of a soft robot with a biologically inspired and biomorphic visual appearance and form, set in a neutral photo studio:1.3)), (((photo studio setting:1.2))), ((natural and realistic lighting), (soft-bodied robot), ((Soft materials)), pliable, (biomorphic form)), (biomorphic robot), (organic form), (organic surface texture), (((soft natural organism))), asymetrical, bulbous, rugged, arciform, sweeping, annular, undulating and irregular contours, photography, RAW, DSLR, high resolution, HiRes, High quality, (natural colors)

Negative prompt: (plastic), ((metal)), painting, drawing, cartoon, rendering, 3D, computer graphics, out of proportions, (saturated colors) , saturated, blurry, ((low resolution)), LoRes, (bad quality)

Steps: 50, Sampler: Euler a, CFG scale: 9, Seed: -1, Size: 512x512, Model hash: 6ce0161689, Model: v1-5-pruned-emaonly, Version: v1.6.0

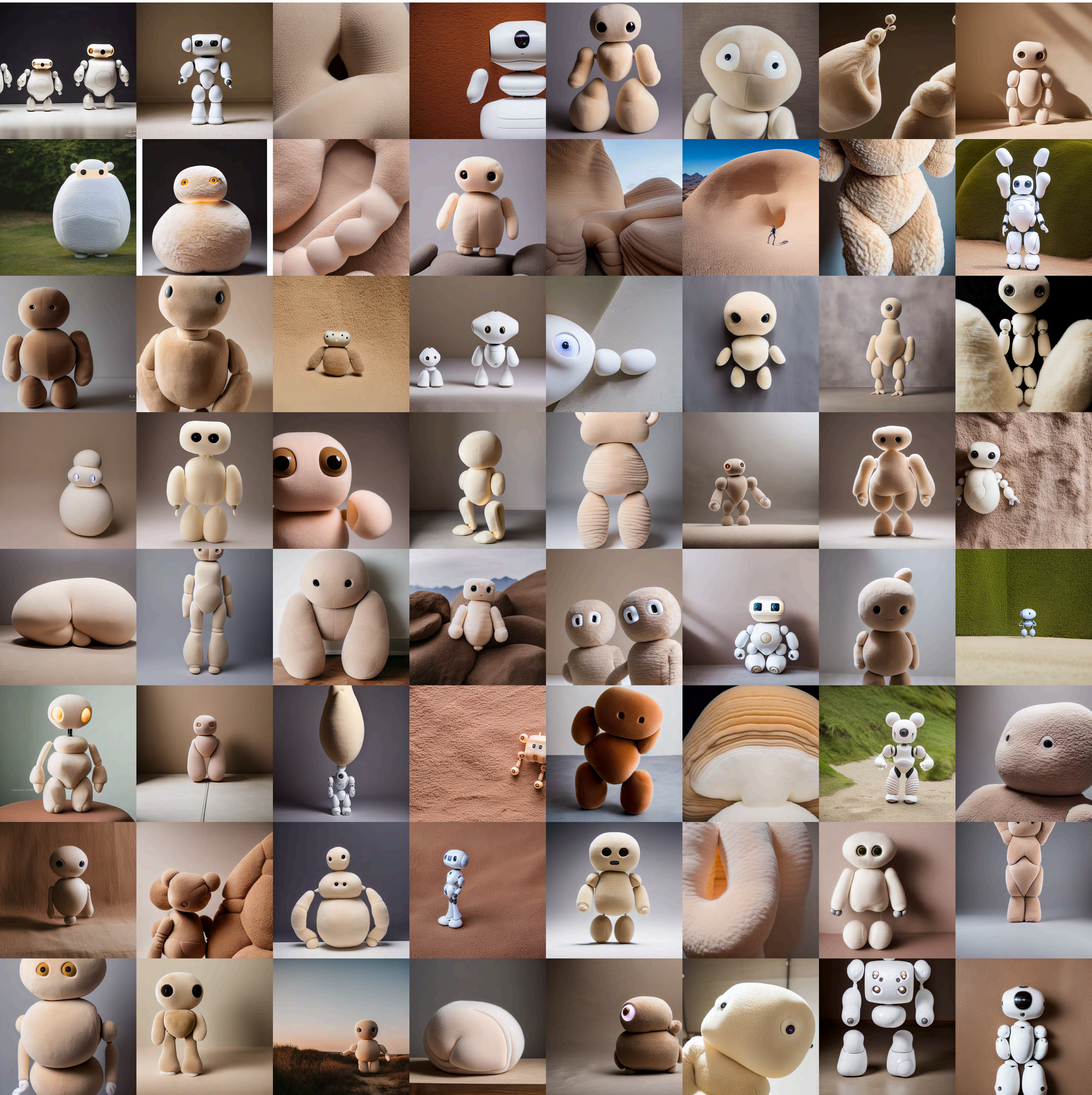

Prompt Experiments:
64 TTI generated images - 4th round

SETTINGS

Prompt: ((full-body image of a soft robot with a biologically inspired and biomorphic visual appearance, form and surface texture, set in a neutral photo studio:1.3)), (((photo studio setting:1.2))), ((natural and realistic lighting), (soft-bodied robot:1.5), ((Soft materials)), pliable, (biomorphic form)), (biomorphic robot), (organic form), (organic surface:2), (((soft natural organism))), asymetrical, bulbous, rugged, arciform, sweeping, annular, undulating and irregular contours, photography, RAW, DSLR, high resolution, HiRes, High quality

Negative prompt: (plastic), ((metal)), painting, drawing, cartoon, rendering, 3D, computer graphics, out of proportions, (saturated colors) , saturated, blurry, ((low resolution)), LoRes, (bad quality)

Steps: 50, Sampler: Euler a, CFG scale: 9, Seed: -1, Size: 512x512, Model hash: 6ce0161689, Model: v1-5-pruned-emaonly, Version: v1.6.0

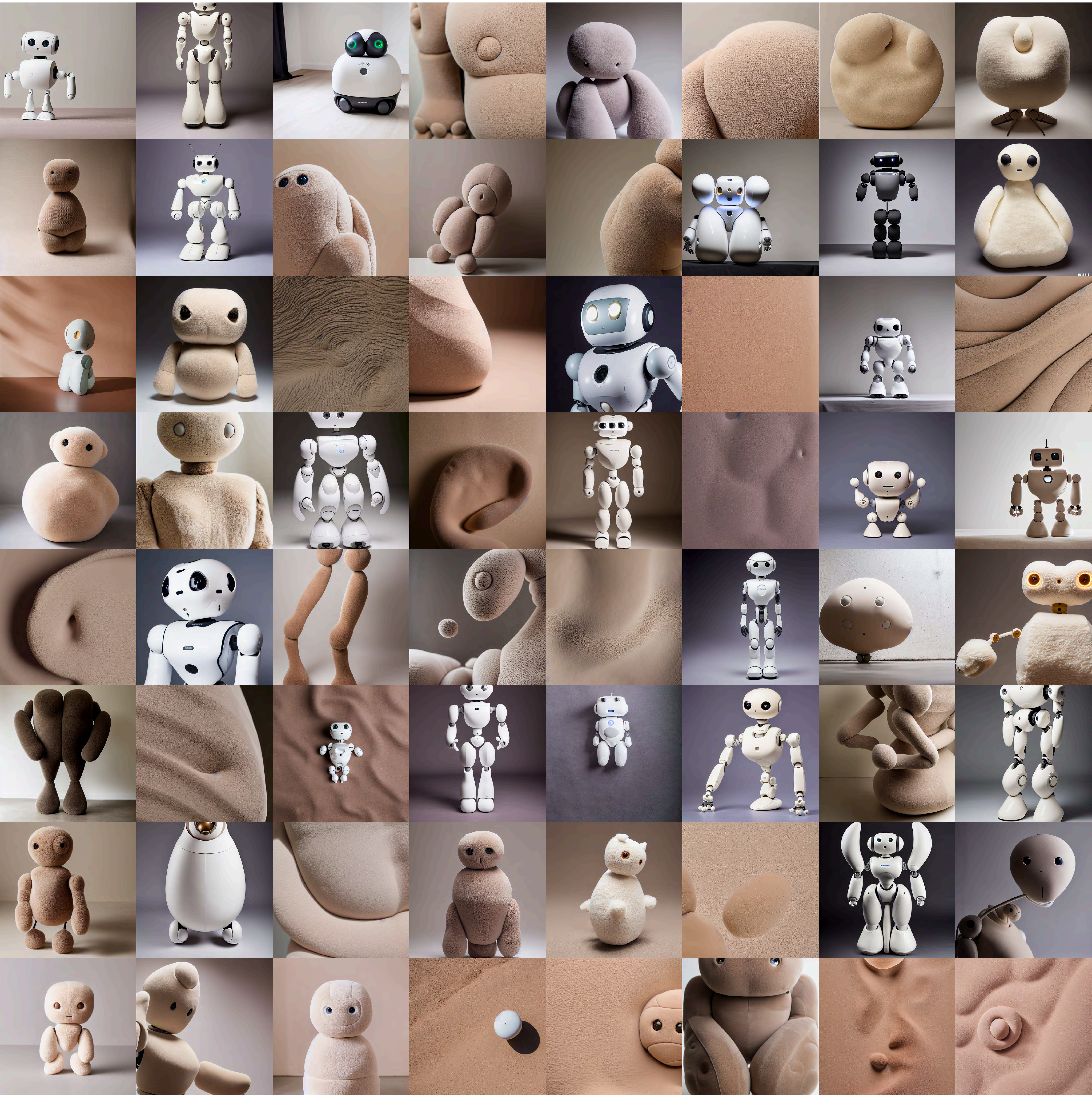

Prompt Experiments:
64 TTI generated images - 5th round

SETTINGS

Prompt: ((full-body image of a soft robot with a biologically inspired and biomorphic visual appearance, form and surface texture, set in a photo studio:1.3)), (photo studio setting:1.5), (soft robot made from organic looking material:2), ((Soft materials)), pliable, (biomorphic form)), (robot surface has vivid organic polychromatic coloring and nuances:1.3), (biomorphic robot), (organic form), (organic surface), (((soft natural organism))), asymetrical, bulbous, rugged, arciform, sweeping, annular, undulating and irregular contours, photography, RAW, DSLR, high resolution, HiRes, High quality

Negative prompt: (plastic), ((metal)), painting, drawing, cartoon, rendering, 3D, computer graphics , saturated, blurry, ((low resolution)), LoRes, (bad quality)

Steps: 50, Sampler: Euler a, CFG scale: 9, Seed: -1, Size: 512x512, Model hash: 6ce0161689, Model: v1-5-pruned-emaonly, Version: v1.6.0

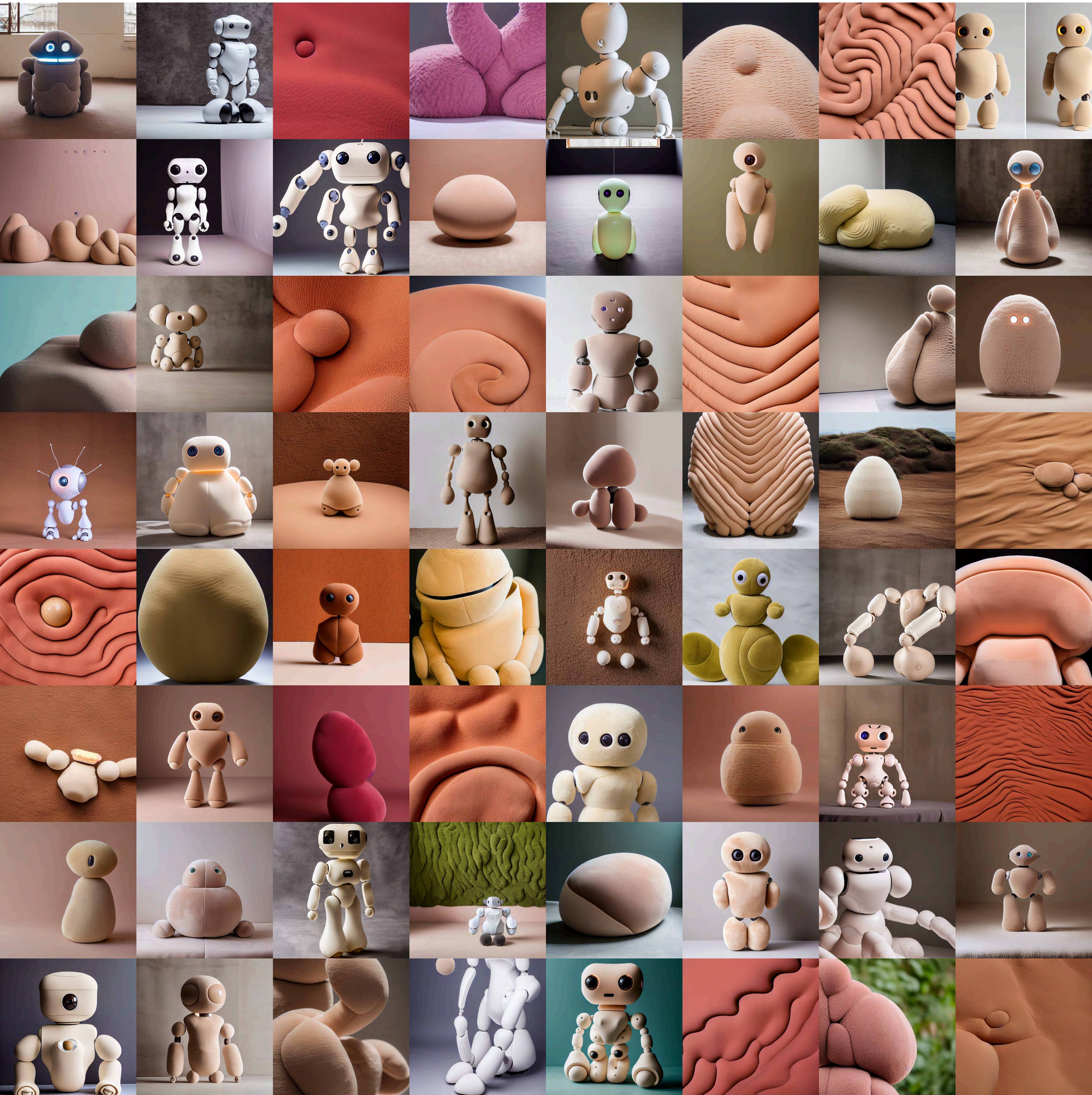

Supplement: Supplementary file 1 [file DataSheet2.pdf]
