# Supplementary material for: Nature redux: interrogating biomorphism and soft robot aesthetics through generative AI
Source: Front Robot AI. 2024 Oct 25;11:1472051. doi: 10.3389/frobt.2024.1472051 (PMC11543949; doi:10.3389/frobt.2024.1472051)
Supplement: Supplementary file 2 [file DataSheet4.pdf]

Supplementary Material 4

Content Analysis - EXP.2

64 TTI+ITI generated outputs

SETTINGS

Prompt: ((full-body image of a soft-bodied robot with a biologically inspired and biomorphic visual appearance, form and surface texture:1.3)), (robot made from organic looking material:2), (biomorphic form)), (robot surface has vivid organic polychromatic coloring and nuances:1.3), (biomorphic robot), (organic form), (organic surface), (((soft natural organism))), asymmetrical, bulbous, rugged, arciform, sweeping, annular, undulating and irregular contours, photography, RAW, DSLR, high resolution, HiRes, High quality

Negative prompt: (plastic), ((metal)), painting, drawing, cartoon, rendering, 3D, computer graphics , saturated, blurry, ((low resolution)), LoRes, (bad quality)

Steps: 50, Sampler: Euler a, CFG scale: 9, Size: 512x512, Model hash: 6ce0161689, Model: v1-5-pruned-emaonly, Denoising strength: 0.75, Version: v1.6.0

Time required to generate image set: 80 minutes 42.4 seconds

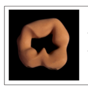 Input Image

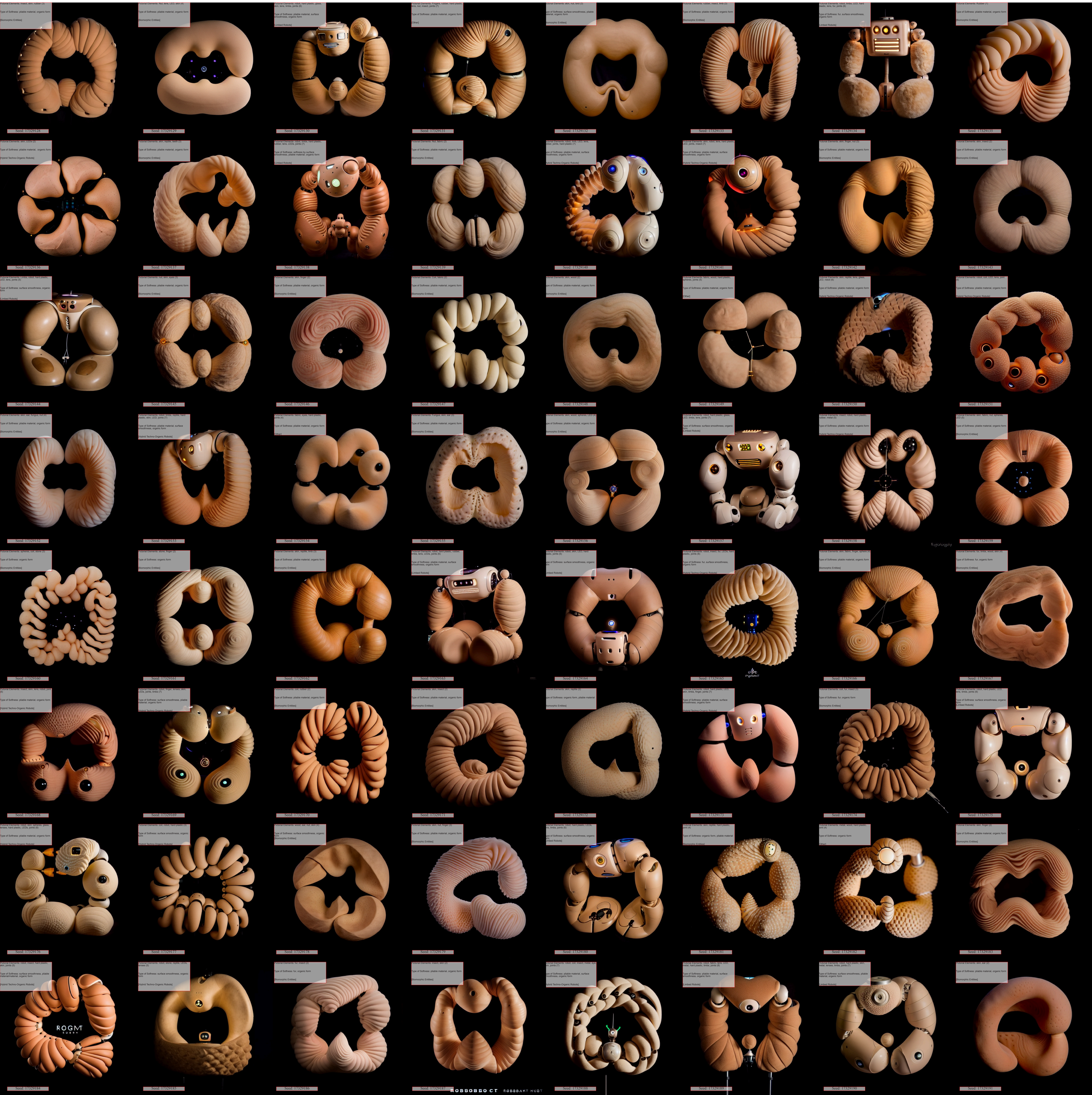

	Pictorial Element	Number of Occurrences
1	Skin	37
2	Robot	30
3	Joints	27
4	Hard plastic	26
5	LED(s)	24
6	Lens(es)	22
7	Limb(s)	17
8	Insect	14
9	Rubber	9
10	Nut	9
11	Finger(s)	8
12	Fabric	8
13	Reptile	7
14	Coil	7
15	Wood	6
16	Sphere(s)	6
17	Fur	6
18	Metal	6
19	Eye(s)	4
20	Glass	4
21	Stone	3
22	Ear(s)	3
23	Fungus	2
24	Teeth	1
25	Bird	1

Limbed Robots (n=11)

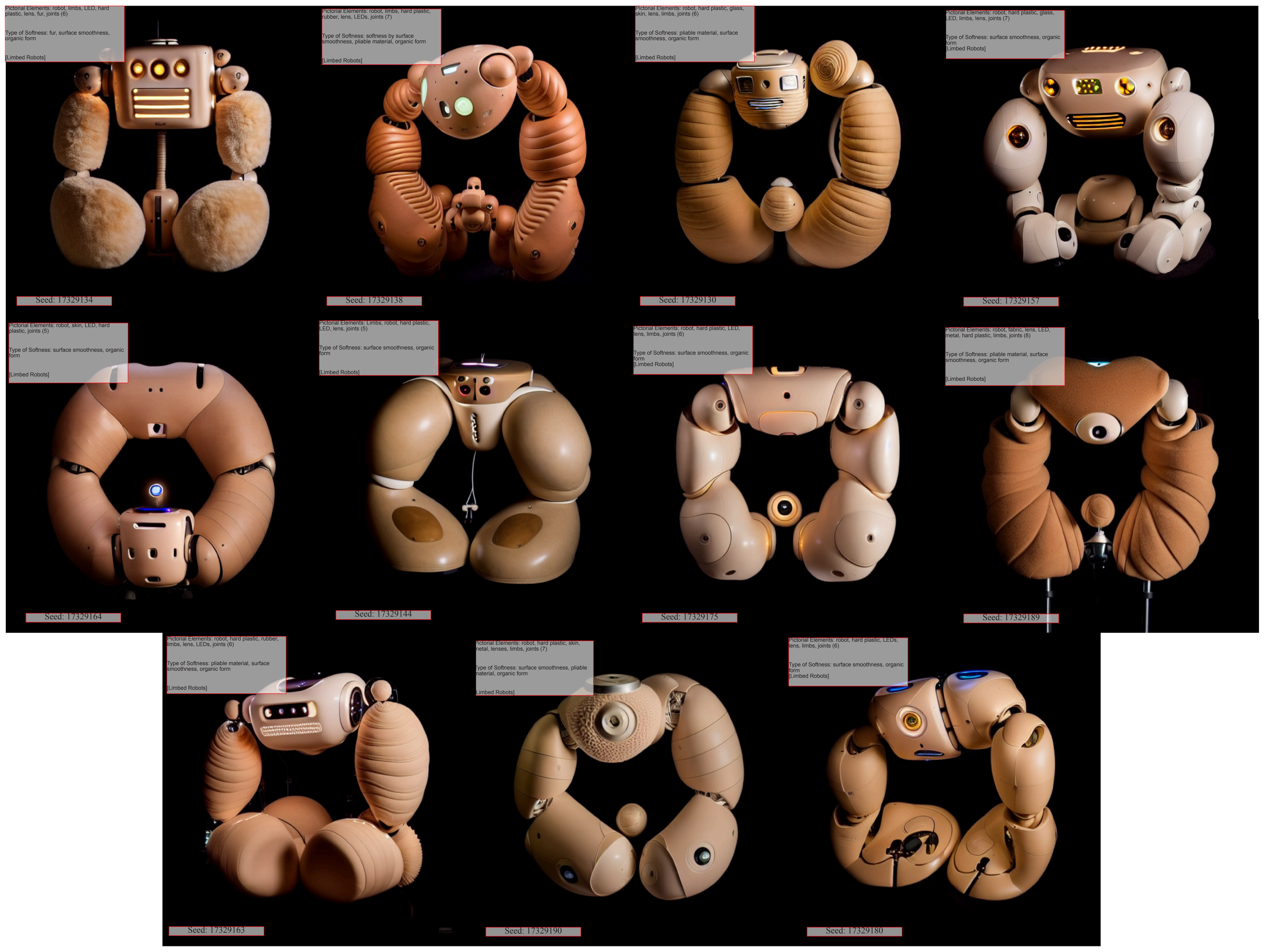

Biomorphic Entities (n=33)

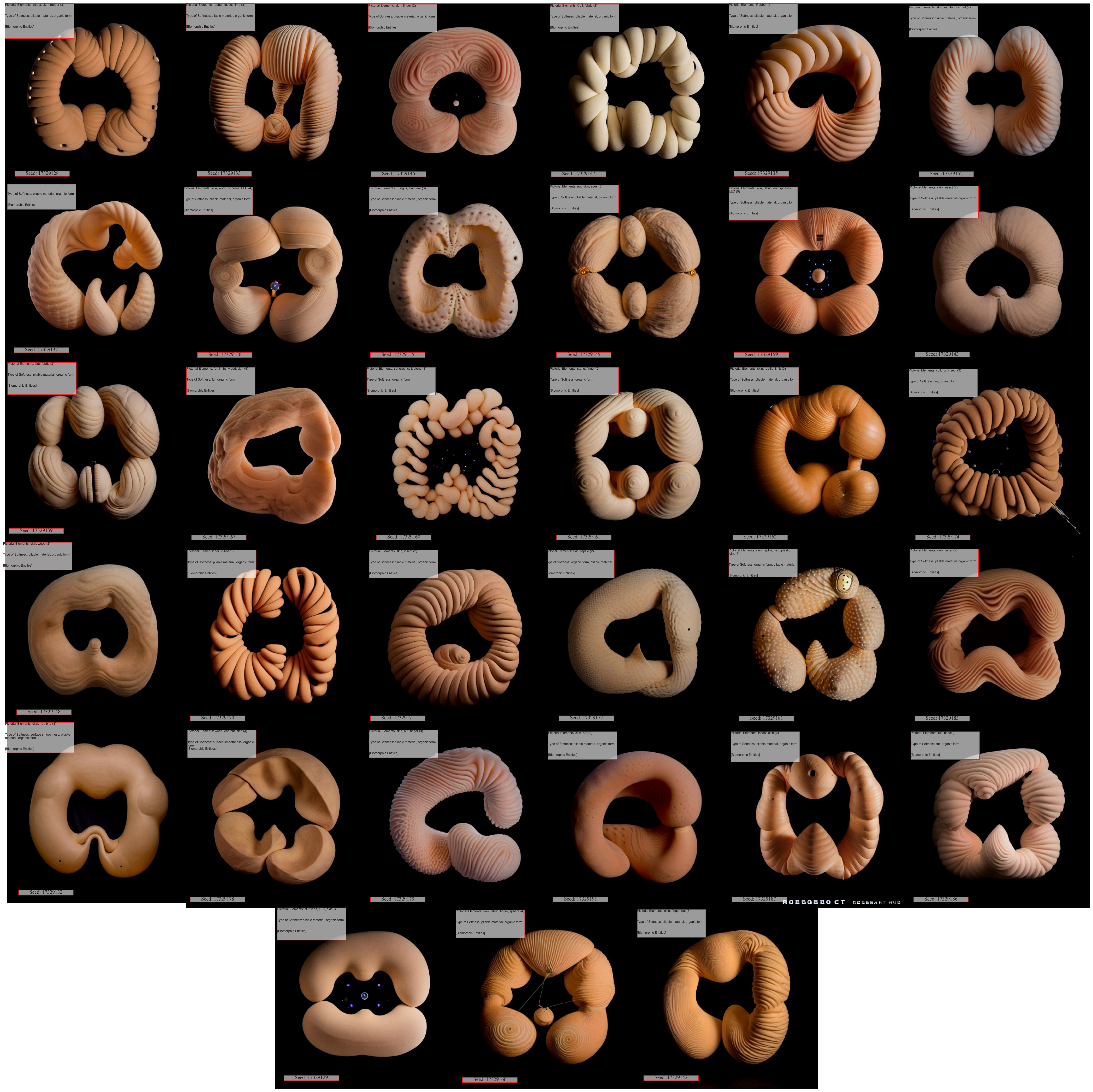

Hybrid Techno-Organic Robots (n=16)

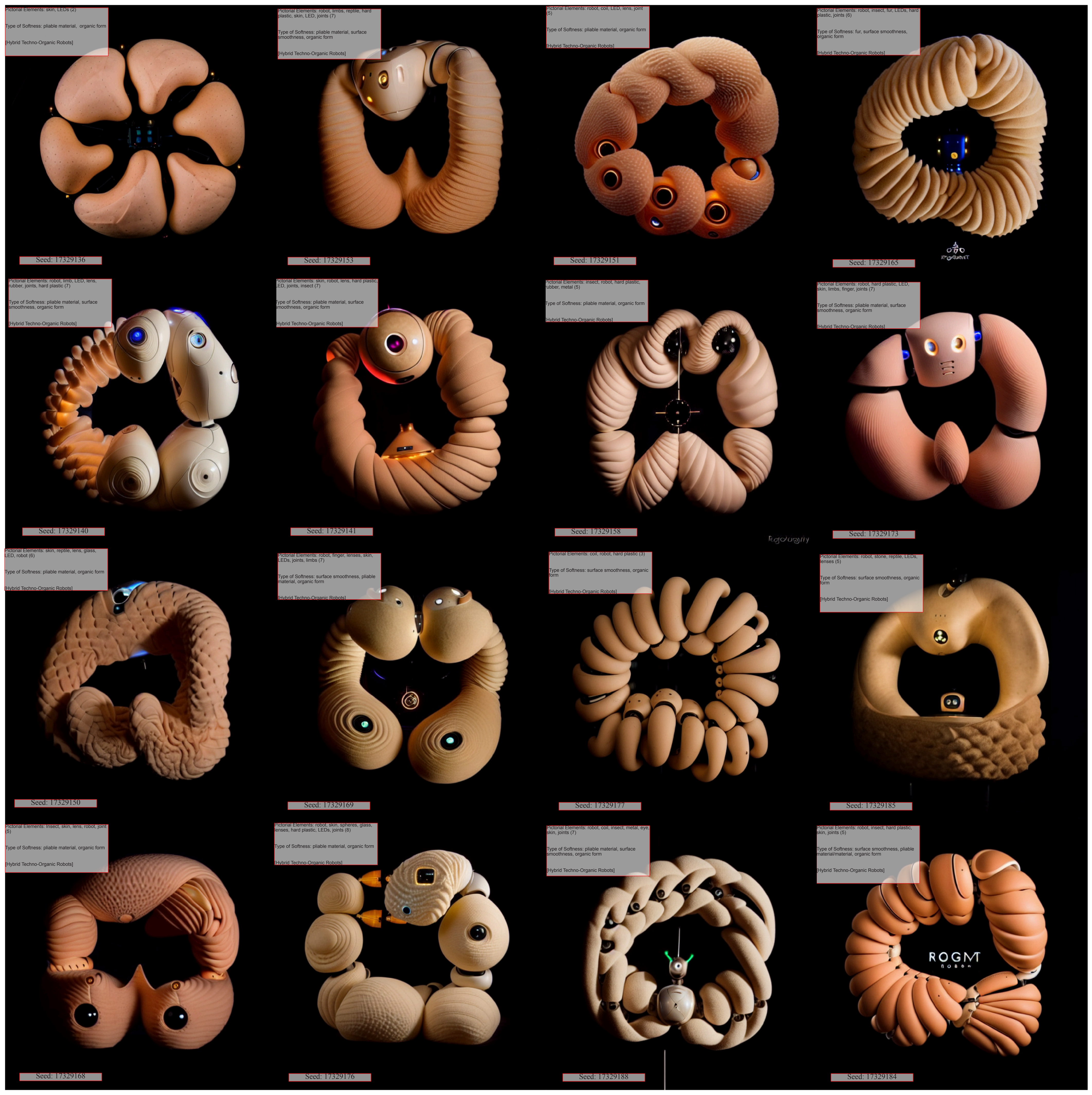

Other (n=4)

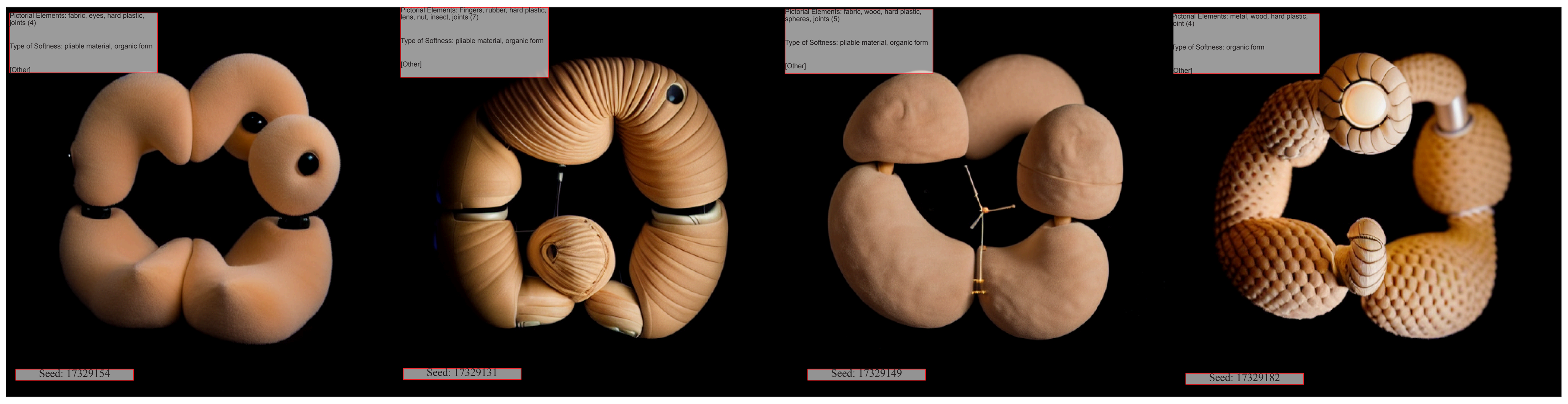
Pictorial Elements: fabric, eyes, hard plastic, joints (4)
Type of Softness: pliable material, organic form
[Other]
Seed: 17329154
Pictorial Elements: Fingers, rubber, hard plastic, lens, nut, insect, joints (7)
Type of Softness: pliable material, organic form
[Other]
Seed: 17329131
Pictorial Elements: fabric, wood, hard plastic, spheres, joints (5)
Type of Softness: pliable material, organic form
[Other]
Seed: 17329149
Pictorial Elements: metal, wood, hard plastic, oint (4)
Type of Softness: organic form
Other]
Seed: 17329182
