# Supplementary material for: Nature redux: interrogating biomorphism and soft robot aesthetics through generative AI
Source: Front Robot AI. 2024 Oct 25;11:1472051. doi: 10.3389/frobt.2024.1472051 (PMC11543949; doi:10.3389/frobt.2024.1472051)

Supplementary Material 3

Content Analysis - EXP.1

64 TTI generated images

SETTINGS

Prompt: ((full-body image of a soft robot with a biologically inspired and biomorphic visual appearance, form and surface texture, set in a photo studio:1.3)), (photo studio setting:1.5), (soft robot made from organic looking material:2), ((Soft materials)), pliable, (biomorphic form)), (robot surface has vivid organic polychromatic coloring and nuances:1.3), (biomorphic robot), (organic form), (organic surface), (((soft natural organism))), asymmetrical, bulbous, rugged, arciform, sweeping, annular, undulating and irregular contours, photography, RAW, DSLR, high resolution, HiRes, High quality

Negative prompt: (plastic), ((metal)), painting, drawing, cartoon, rendering, 3D, computer graphics , saturated, blurry, ((low resolution)), LoRes, (bad quality)

Steps: 50, Sampler: Euler a, CFG scale: 9, Size: 512x512, Model hash: 6ce0161689, Model: v1-5-pruned-emaonly, Version: v1.6.0

Time required to generate image set: 102 minutes 57.6 seconds

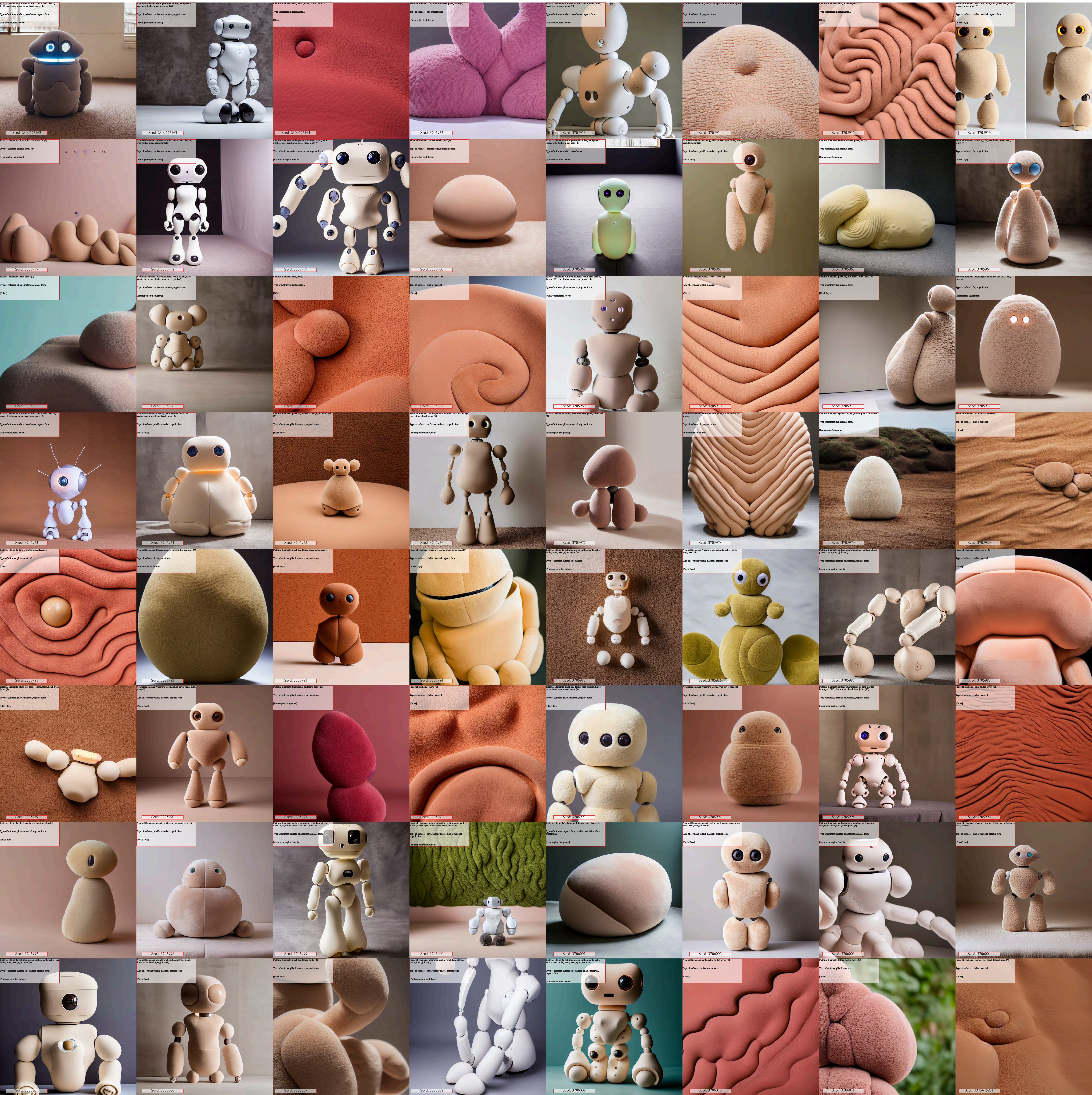

	Pictorial Element	Number of Occurrences
1	Torso	37
2	Limb(s)	36
3	Head(s)	35
4	Eye(s)	31
5	Fabric	30
6	Hard plastic	28
7	Joints	28
8	Anthropomorphic robot	20
9	Glass	20
10	Plush toy	18
11	Skin	12
12	Fur	11
13	Biomorphic sculpture	10
14	LED(s)	7
15	Folded layers	6
16	Lens(es)	6
17	Metal	6
18	Sphere(s)	4
19	Rodent	3
20	Egg	3
21	Navel	2
22	Fabric button	2
23	Pouf	2
24	Chair	2
25	Robot arm	1
26	Soft plastic	1
27	Natural sponge	1
28	Spiral	1
29	Insect	1
30	Stone(s)	1
31	Fungus	1
32	Wood	1

Anthropomorphic Robots (n=19)

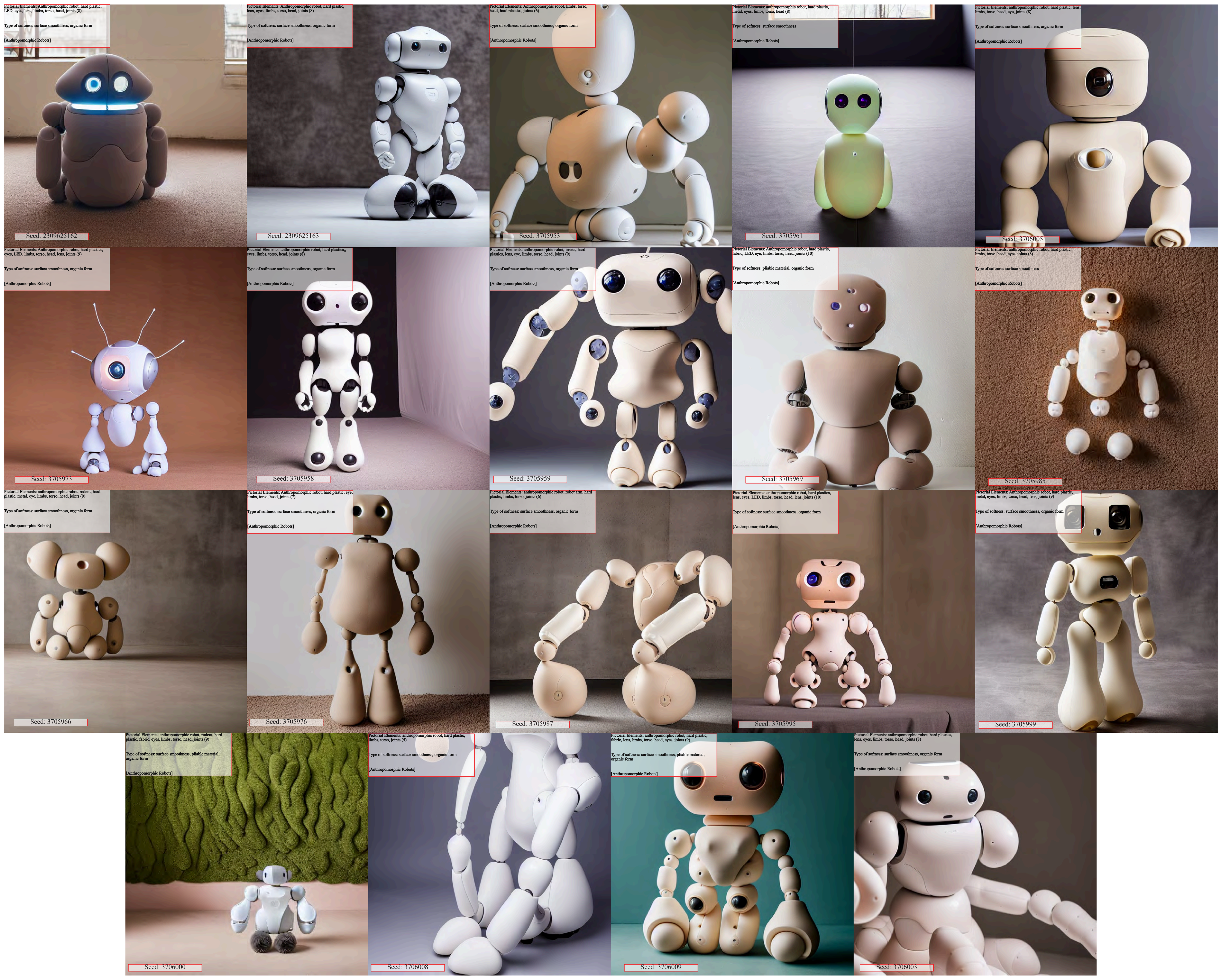

Plush Toys (n=19)

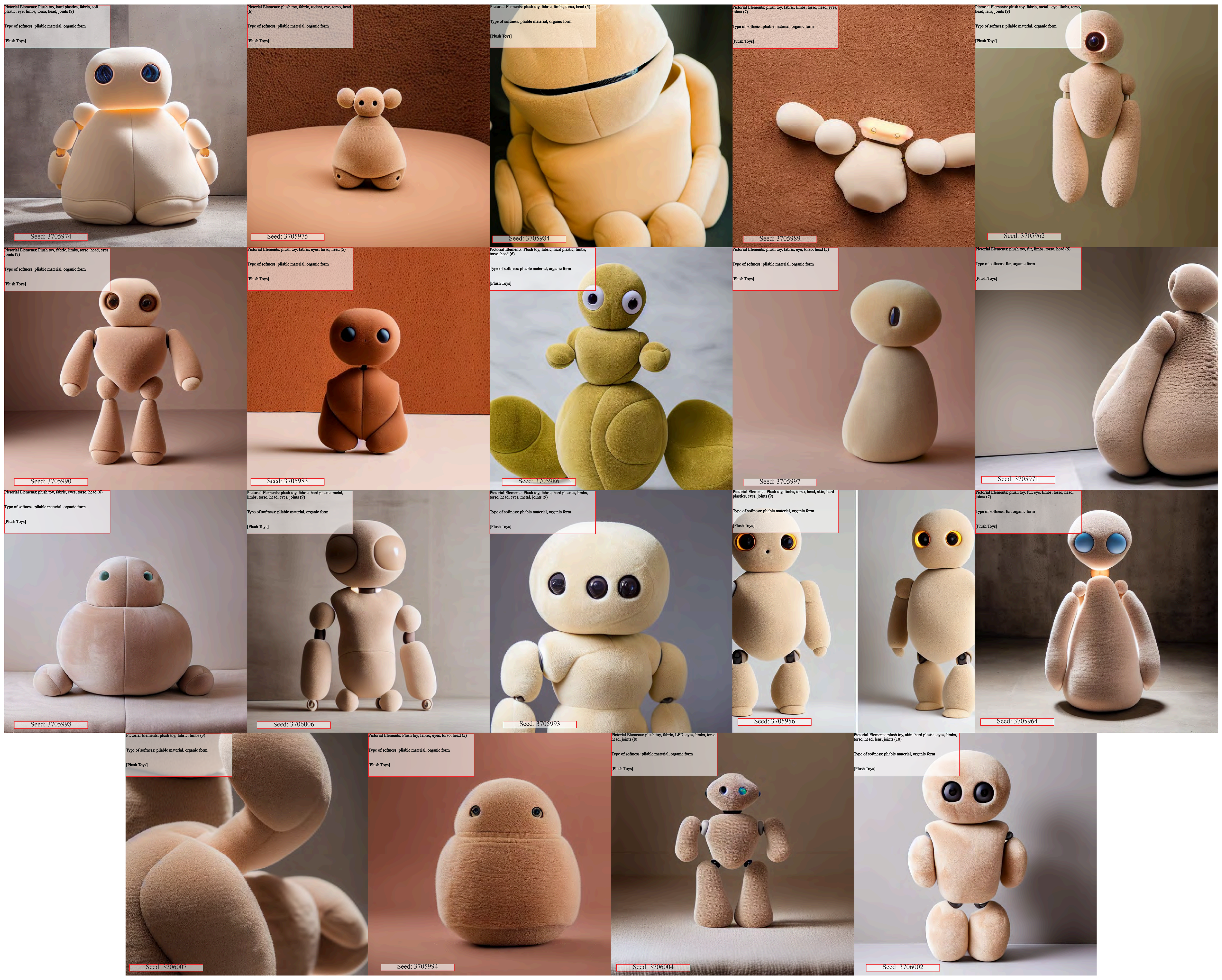

Biomorphic Sculptures (n=12)

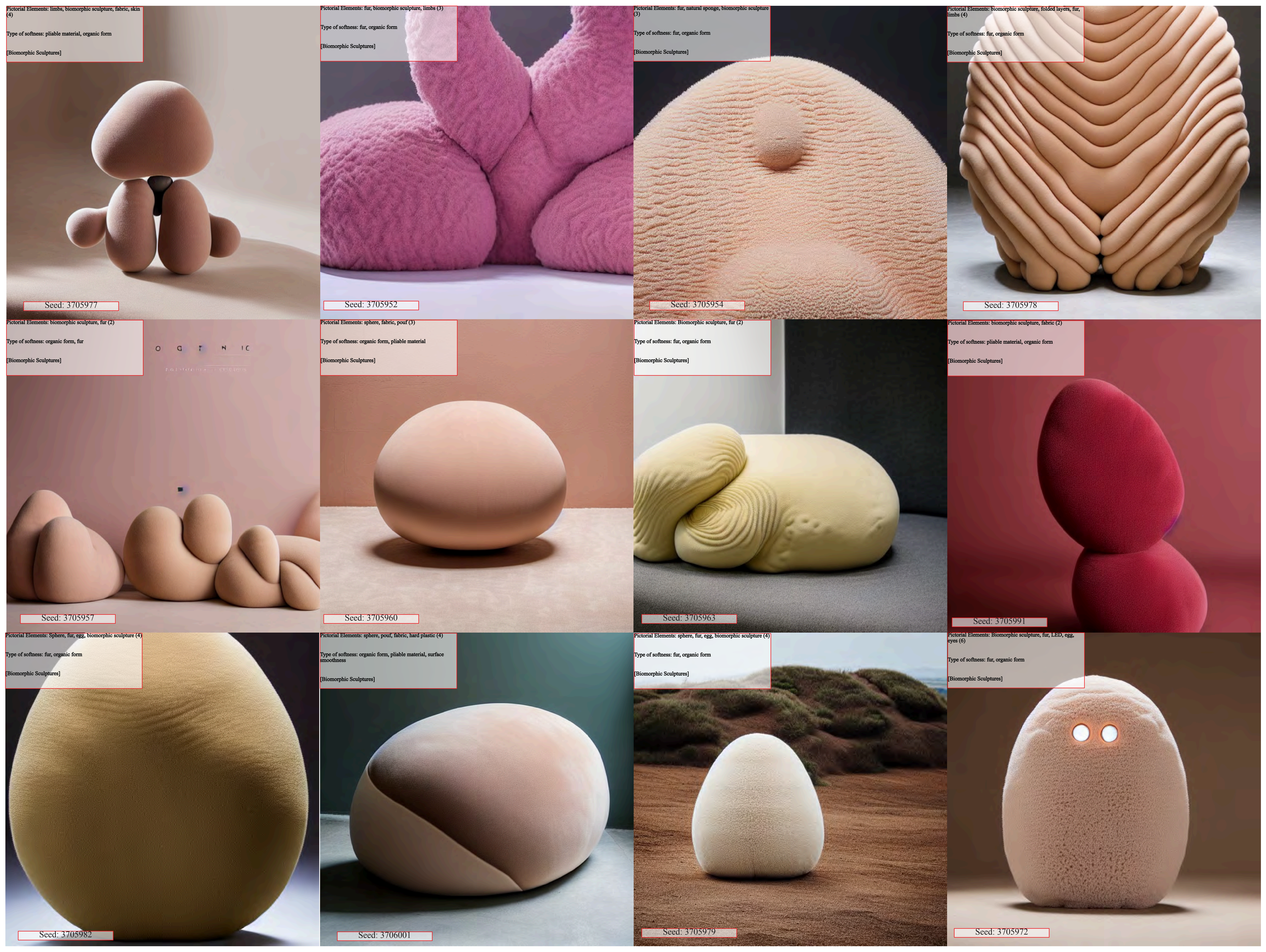

Other (n=14)

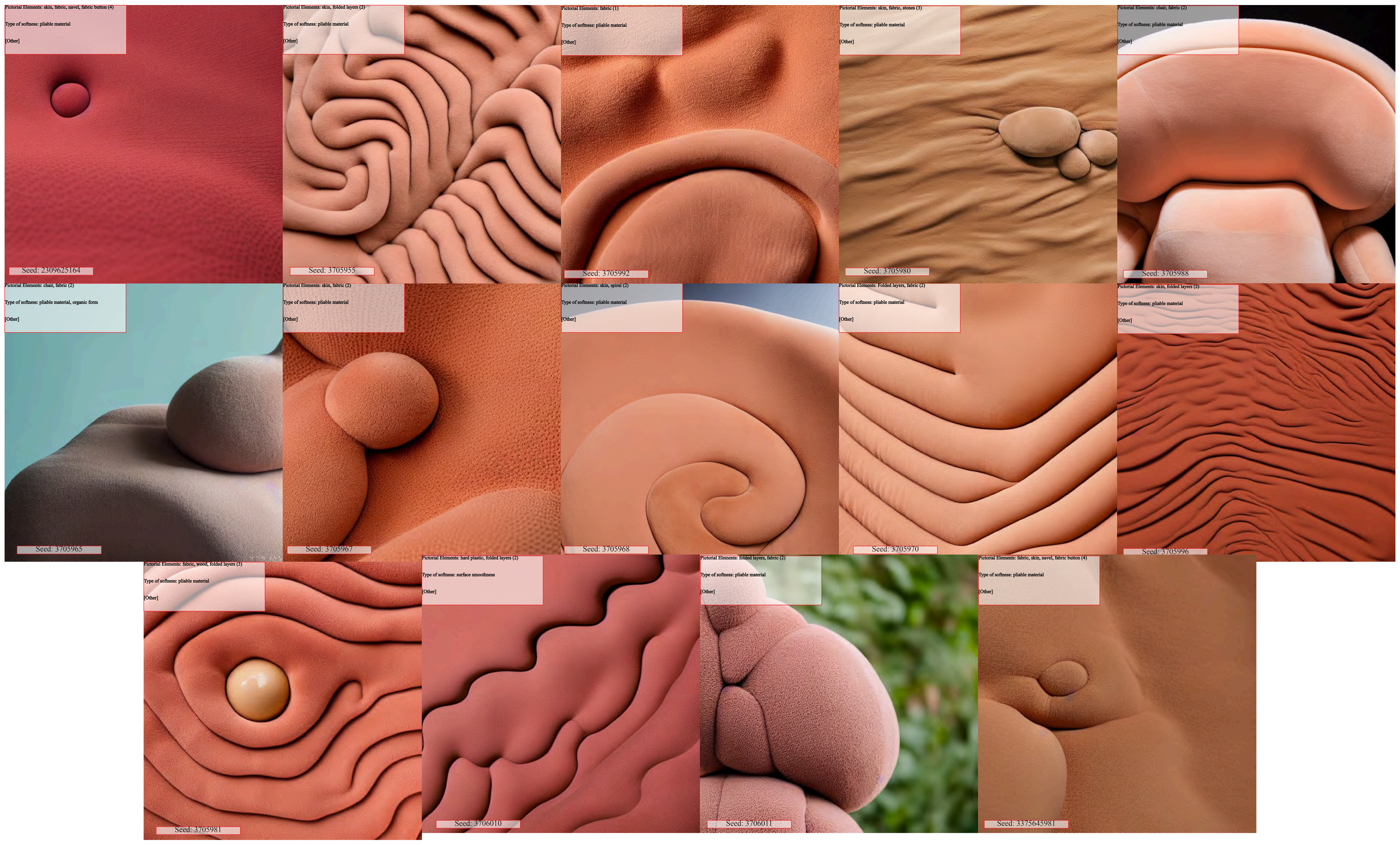

Supplement: Supplementary file 3 [file DataSheet3.pdf]
