# Supplementary material for: Nature redux: interrogating biomorphism and soft robot aesthetics through generative AI
Source: Front Robot AI. 2024 Oct 25;11:1472051. doi: 10.3389/frobt.2024.1472051 (PMC11543949; doi:10.3389/frobt.2024.1472051)
Supplement: Supplementary file 4 [file DataSheet1.pdf]

Supplementary Material 1

“Nature Redux: Interrogating Soft Biomorphism and Soft Robot Aesthetics Through Generative AI”

Mads Bering Christiansen, Ahmad Rafsanjani, and Jonas Jørgensen

Configuration of Stable Diffusion

We generated images using the *v1-5-pruned-emaonly checkpoint* (Rombach & Esser, n.d.) in *Experiment 1* and *Experiment 2*. The checkpoint refers to pre-trained model weights that are trained on sets of specific styles or genres of images. We chose the v1-5 checkpoint specifically because it is a broad general-purpose model that can be used for generating photo-realistic images of many different types of inputs (Rombach & Esser, n.d.). We set the *seed* value for noise generation to -1 (random seed) in our experiments. The *sampling method* and *sampling steps* were also configured. The sampling method refers to the algorithm used to go from random noise to a generated image, and the sampling steps to the number of iterations in the process. These parameters both affect the overall visual style, image contents, and image quality. Based on prior work (Bagu, 2023; McCormick, 2023; Patel, 2023; RunDiffusion, 2023; Stable Diffusion Art, 2023a), we chose the *Euler Ancestral* sampling method running 50 sampling steps. The Euler Ancestral sampling method adds additional randomness to each denoising step in Stable Diffusion, thereby generating a broader variation in the visual outputs (Stable Diffusion Art, 2023a). This has drawbacks in terms of less consistency between each sampling step but can be a useful sampling method when searching for inspiration (McCormick, 2023), or in our case seeking to explore an extensive variety of visual outputs. Setting the sampling steps to 50 caused slow generation speeds but resulted in highly detailed visual outputs, allowing for close inspection in our content analyses and categorizations of the outputs.

In *Experiment 1* that used TTI generative AI, we adjusted how closely the AI conformed to the provided text prompts. This is determined by the *classifier-free guidance* (*CFG*) scale in the range of 1-30. Increasing the CFG value makes Stable Diffusion conform closely to text prompts, however at the cost of lower overall image quality. On the contrary, lowering the CFG scale increases the image quality while drastically steering away from the provided text prompts. Following the recommendations from Getimg.ai (n.d.), we set the CFG scale value to 9, striking a compromise between image quality and flexibility for unexpected outcomes.

In *Experiment 2*, in which we combined TTI and ITI generation, we used the same CFG scale value as in *Experiment 1* for overall consistency. Furthermore, we adjusted the *denoising strength* in *Experiment 2*. Ranging from 0 to 1, the denoising strength value determines the amount of noise added during the reverse process of recovering a denoised image from the “noisy” or diffused version. A denoising

strength of 0 results in an output completely mirroring the input, while a setting of 1 leads to only noise, generating an output fully unrelated to the input. Following recommendations for how to generate images that differed from their input source but were not entirely unrelated (Stable Diffusion Art, 2023b), we set the denoising strength to 0.75.

References

Bagu, B. (2023, July 11). Sampling Steps in Stable Diffusion: Finding A Balance With Examples. *Medium*. https://medium.com/@braden.bagu/optimizing-sampling-steps-in-stable-diffusion-a-practical-guide-with-examples-5716dc036fa1

Getimg.ai. (n.d.). *Guide to Stable Diffusion CFG scale (guidance scale) parameter*. Retrieved 3 November 2023, from https://getimg.ai/guides/interactive-guide-to-stable-diffusion-guidance-scale-parameter

McCormick, C. (2023, April 11). *Choosing a Sampler for Stable Diffusion · Chris McCormick*. https://mccormickml.com/2023/04/11/choosing-stable-diffusion-sampler/

Patel, A. (2023, May 19). Sampling Methods for Stable Diffusion: Samplers Comparison. *ADESIGNE*. https://adesigne.com/artificial-intelligence/sampling-methods-for-stable-diffusion-samplers-comparison-guide/

Rombach, R., & Esser, P. (n.d.). *Runwayml/stable-diffusion-v1-5 · Hugging Face*. Retrieved 27 March 2023, from https://huggingface.co/runwayml/stable-diffusion-v1-5

RunDiffusion. (2023, May 26). *Sampling Methods*. RunDiffusion Docs. https://learn.rundiffusion.com/sampling-methods/

Stable Diffusion Art. (2023a, March 28). *Stable Diffusion Samplers: A Comprehensive Guide*. https://stable-diffusion-art.com/samplers/

Stable Diffusion Art. (2023b, November 24). *Beginner’s guide to inpainting (step-by-step examples)—Stable Diffusion Art*. https://stable-diffusion-art.com/inpainting_basics/
